# Oxygen and pH fluxes in shallow bay habitats: Evaluating the effectiveness of a macroalgal forest restoration

**DOI:** 10.1111/jpy.13520

**Published:** 2024-11-18

**Authors:** Cristina Galobart, Cèlia Sitjà, Sònia de Caralt, Jorge Santamaría, Alba Vergés, Jordi Boada, Emma Cebrian

**Affiliations:** ^1^ Centre d'Estudis Avançats de Blanes (CEAB‐CSIC) Blanes Spain; ^2^ Institut d'Ecologia Aquàtica (IEA), Universitat de Girona (UdG) Girona Spain

**Keywords:** ecosystem functioning, in situ benthic incubations, macroalgal primary production, restoration success, shallow marine forests

## Abstract

Marine macroalgae are important primary producers in coastal ecosystems. Within sheltered and shallow bays in the Mediterranean, various Fucalean macroalgae and seagrasses coexist, creating habitats of high ecological importance. These habitats have historically suffered from various disturbances, and on this basis, active restoration actions have been proposed as potential solutions for their recovery. Here, we assessed the restoration success of a 10‐year restored macroalgal forest by evaluating the recovery in terms of oxygen and pH fluxes and comparing those data with those of a healthy marine forest and a degraded habitat counterpart. We estimated the overall changes in dissolved oxygen and pH using light and dark community in situ incubations. We also determined the biomass and composition of macroalgal and macroinvertebrate compartments of each assemblage. During light incubations, the healthy and restored forest assemblages showed similar average net oxygen production, 5.7 times higher than in the degraded one, and a greater increase in pH. More than 95% of the incubated biomass corresponded to macroalgal and seagrass species. The restored forest showed a six‐fold increase in biomass, most likely being responsible for the recovery of primary production. This work provides empirical evidence that the restoration of a single structural species, once successful in the early stages, can yield positive results by recovering processes such as primary production and dark respiration. Moreover, these results showcase differences in ecosystem functions between healthy (either mature or restored) and degraded habitats, highlighting the importance of protecting and preserving coastal marine forests.

**Abbreviations**
ANOVA: analysis of variance
GPP: gross primary production
nMDS: nonmetric multidimensional scaling
NPP: net primary production
R: dark respiration

## INTRODUCTION

Marine macrophytes, including macroalgae and seagrasses, are the main drivers of primary production in temperate coastal regions (Mann, 1973; Thayer et al., 1975). They underpin entire ecosystems by generating large amounts of oxygen and synthesizing organic matter via photosynthesis (Duarte, 2017; Pessarrodona et al., 2022). These macrophytes also provide habitat and shelter for numerous organisms (Cheminée et al., 2013; Christie et al., 2009; Teagle et al., 2017) and are particularly important for human societies, delivering a wealth of ecosystem functions and services, such as the protection of coastlines against waves, reduction of nutrient loads, and support of commercial fisheries (Bennett et al., 2016; Costanza et al., 1997; Eger et al., 2023; Mtwana Nordlund et al., 2016).

In Europe, among the inherent variability of the coastline, sheltered bays with limited freshwater are known to host vegetated habitats of significant ecological importance. These habitats are protected under the European Habitats Directive (Council Directive 92/43/EEC) and come within the Natura 2000 framework (Annex I: natural habitat types of community interest) under the habitat classification “Large shallow inlets and bays,” code 1160. The benthic communities typical of these areas show a marked zonation with a wide diversity of sediment substrates (European Commission: Directorate‐General for the Environment, 2013). In the Mediterranean Sea, the shallowest substrate of large bays is usually a combination of sand and rocks. This specific zone of the infralittoral is highly protected and has abundant light, allowing different macroalgal and seagrass species to thrive and coexist (Ribera et al., 1997; Sales & Ballesteros, 2009).

Due to the peculiar characteristics of these areas, they have historically sustained a constant human presence and significant urbanization, which has resulted in habitat destruction and seawater pollution (Airoldi & Beck, 2007; Helskini Commission [HELCOM], 2013; Michałek & Kruk‐Dowgiałło, 2016). In addition, other pressures, such as localized eutrophication and overgrazing by sea urchins and fish, have further impacted large macroalgal habitats (i.e., marine forests), leading to habitat degradation (e.g., Iveša et al., 2016; Moy & Christie, 2012; Vergés et al., 2014). A common consequence of these impacts is the replacement of highly complex and structured macroalgal forests with small, mat‐forming algae and, in extreme cases, barren grounds (Filbee‐Dexter & Wernberg, 2018; Minguito‐Frutos et al., 2023; Vergés et al., 2014). These shifts can cause alterations and losses in some ecosystem functions and processes, such as food webs and energy flows (Vergés et al., 2019) and primary production (Rocha et al., 2015). The persistence in time of these losses depends on multiple factors, including the intensity and duration of the impact(s) that caused them, together with the existence of nearby populations with the potential to act as a source of new recruits (e.g., Ling et al., 2010; Reed et al., 2004). However, despite some documented examples of natural recovery of marine forests, they often fail to recover even after the removal of the initial impact (e.g., Díez et al., 2009; Pinedo et al., 2013). To this effect, ecological restoration has emerged as a proactive tool with the potential to reverse the loss and degradation of these ecosystems (Aronson & Alexander, 2013; Suding et al., 2015).

Considerable advances have been made in terrestrial ecosystems, where restoration efforts have not only focused on improving vegetation structure and species diversity but also on rehabilitating lost functions and services, ensuring that these ecosystems deliver their former or potential benefits (Perring et al., 2015; Suding et al., 2015). Meanwhile, marine restoration, particularly marine forest restoration, has also gained traction in recent years (Eger et al., 2022), with numerous case studies detailing protocols and methods before and during restoration actions (e.g. de Caralt et al., 2023; Fredriksen et al., 2020; Layton et al., 2021; see Cebrian et al., 2021 for a review). However, literature related to long‐term monitoring and success evaluation in marine forest restoration remains globally sparse (Earp et al., 2022; Eger et al., 2022), with few studies addressing species diversity recovery (Bianchelli et al., 2023; Marzinelli et al., 2016) or assessing ecosystem functioning (Galobart et al., 2023).

A key process describing ecosystem functioning is the contribution to primary production, as it forms the foundation of most food webs and ecosystems across the globe (Heimann & Reichstein, 2008; Leith & Whittaker, 1975). In situ incubations have been suggested as a suitable method to explore the metabolic fluxes (i.e., production and dark respiration rates) of macroalgal benthic habitats (e.g., Golléty et al., 2008; Miller et al., 2009; Rodgers et al., 2015). Metabolic assessments have mainly focused on a single structural species (e.g., Abdullah & Fredriksen, 2004; White et al., 2021), although some examples considering the complete community are available (e.g., Bordeyne et al., 2020; Edwards et al., 2020; Miller et al., 2009; Peleg et al., 2020). From the perspective of ecological restoration and to understand the production and respiration fluxes of these communities as a whole, the focus lies not only on the metabolism of the restored structural species itself but also on that of all associated organisms (other photosynthetic and heterotrophic species). Therefore, in situ community changes in oxygen and pH may, to some extent, provide realistic and valuable information about the recovery of ecosystem processes in restored habitats, since they offer information about photosynthetic activity and the uptake of CO_2_ and other dissolved inorganic carbon forms (Frankignoulle, 1994; Gattuso et al., 1995). In fact, ecosystem primary production and nutrient cycling are fundamental attributes proposed for inclusion in restoration evaluations by the Society of Ecological Restoration (SER) in its “International principles and standards for the practice of ecological restoration” (Gann et al., 2019). Moreover, the European Union recently established the Nature Restoration Law (European Commission: Directorate‐General for the Environment, 2022), which promotes the recovery of biodiversity and the resilience of ecosystems, to maintain or improve the provision of functions and services. Despite the significance of ecosystem‐based metrics, these are often omitted in macroalgal restoration assessments, most likely due to various factors such as the mismatch between the time required for the recovery of overall ecosystem processes (as discussed by Smith et al., 2023) and the short‐term monitoring of most restoration actions (<12 months, Earp et al., 2022).

In the Mediterranean, marine macroalgal forests are characterized by species of the order Fucales, in particular those belonging to the genera *Cystoseira*, *Gongolaria*, and *Ericaria* (Molinari Novoa & Guiry, 2020; Orellana et al., 2019). These species are the most frequently observed and diverse canopy‐forming macroalgae, and they create highly structured and complex habitats (e.g., Ballesteros, 1992). These habitats support high levels of biodiversity (Cheminée et al., 2013; Piazzi et al., 2018; Sales & Ballesteros, 2010) and function in equivalent ways as other macroalgal forests in different regions of the ocean (Wernberg & Filbee‐Dexter, 2019).

In this study, we used in situ benthic incubations to quantify the changes in dissolved oxygen and pH occurring in a Mediterranean‐restored marine forest, taking advantage of a 10‐year restoration action performed in a sheltered and shallow marine bay. We compared the results with a healthy and mature forest from a similar environment and with a degraded assemblage located close to the restoration action. We hypothesized that healthy and mature marine forests support higher net primary production and greater rates of pH increase during light periods compared to degraded habitats. Additionally, we expected that a successful habitat restoration would facilitate the achievement of similar rates to those of the mature forests. Species composition and biomass of macroalgae and macroinvertebrates of each assemblage were also analyzed and compared.

## MATERIALS AND METHODS

### Studied assemblages

**FIGURE 1:**
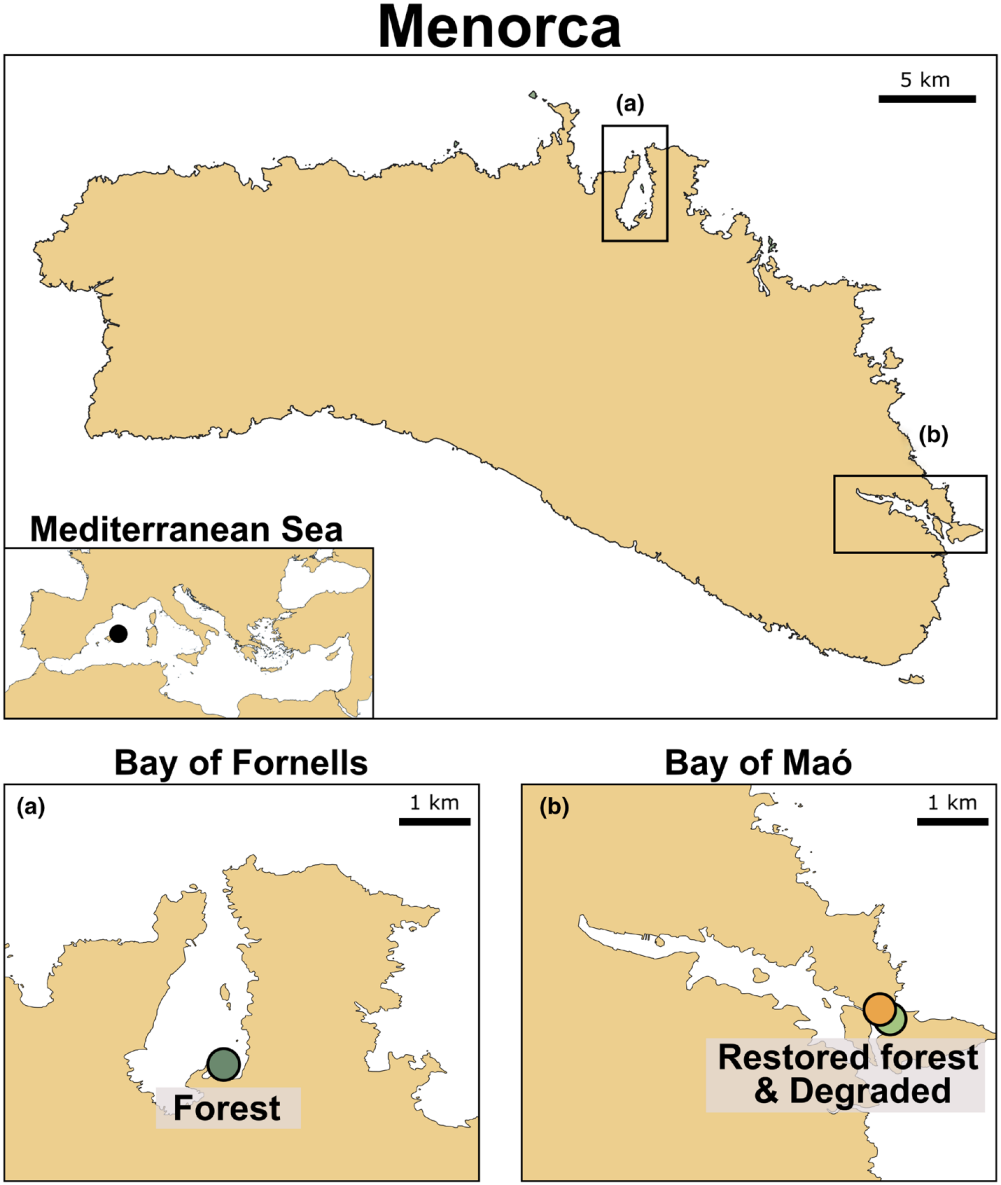
Map of the studied assemblages in Menorca, NW Mediterranean with (a) the Bay of Fornells (b) the Bay of Maó. The former hosts the marine healthy forest, and in the latter, there are the degraded as well as the restored forests.

**FIGURE 2:**
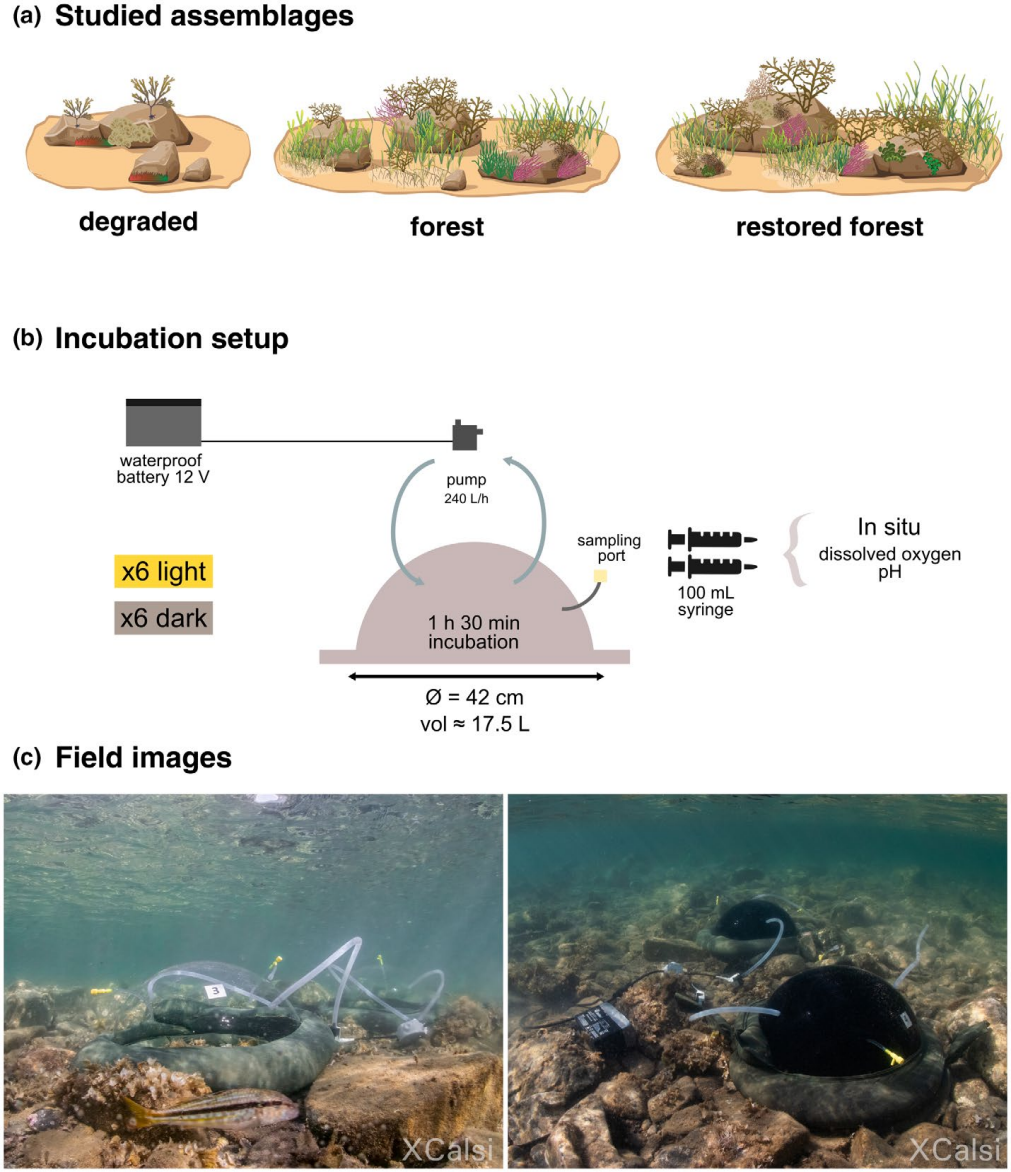
(a) Summarized representation of each assemblage. (b) Schematic diagram of the chamber system. (c) Images of light and dark chambers deployed in situ to estimate dissolved oxygen flux and pH changes of benthic communities. Dome‐shaped chambers were connected to an external pump powered by a waterproof battery. Six transparent and six black incubations of 1 h and 30 min duration were performed at each site. Field images are representative of the degraded assemblage.

*Gongolaria barbata* is a perennial canopy‐forming macroalgae distributed throughout the entire Mediterranean Sea, with moderate annual biomass variation and a peak growth period in spring. However, as with many other Fucalean species, *G. barbata* populations have been declining over the last decades as a consequence of the environmental changes happening on the Mediterranean coasts (Mariani et al., 2019; Orfanidis et al., 2021). Within the Mediterranean, this species is critically endangered, and along the Spanish coasts, there are only two main areas in Menorca (Balearic Islands) where *G. barbata* still exists. In the Bay of Fornells (Figure 1, 40°2′4.46″ N, 4°8′14.03″ E), there are healthy, mature macroalgal forests settled on a patchy substrate of sand and rocks, where the seagrass *Cymodocea nodosa* expands over the sediment and *G. barbata* grows attached to the hard substrate (both on the rocks and on the seagrass rhizomes). The other location is the Bay of Maó (Figure 1, 39°52′48.2″ N, 4°18′30.8″ E), located approximately 50 km south of the Bay of Fornells and sharing similar morphological and environmental features with it (Govern de les Illes Balears, 2007). The structural species *G. barbata* thrived in the area until the 1970s, when it disappeared due to poor water quality (Sales et al., 2011). The construction of a sewage outfall that directly dumped the waste waters into the open sea (Hoyo, 1981) led to the improvement of the water quality, hence removing the impact that initially caused the degradation. However, there was an absence of nearby populations that could supply new recruits naturally, and no recovery of the populations was detected for the following 30 years. In 2011, a restoration action targeted to provide recruits to the area (i.e., recruitment enhancement; see Verdura et al., 2018, for a complete description of the methods used) was implemented. Success was evaluated annually based on the density and size structure distribution of the structural species and the presence of fertile individuals and recruits, which indicated that the restored population was able to self‐maintain (Verdura et al., 2018). Recently, this expansion of the restored population was determined with high‐resolution cartography, showing that the structural species now occupies an area of ~2000 m^2^ compared to the original restoration area of 3.6 m^2^ (Gran et al., 2022). In this area, we assessed the recovery of the oxygen and pH fluxes in the *G. barbata* restored forest. The initial aim of the restoration was to explore and test new methods for restoring Mediterranean Fucalean species, and no metabolic fluxes were measured before the action. As the exact prerestoration assemblage was not available, the assemblage considered in this study as “degraded” was the area adjacent to the restoration site, which features very similar environmental conditions and still lacks *G. barbata*. This area is characterized by low‐complexity algal species and has shown low species diversity and biomass (Galobart et al., 2023). We quantified the overall primary production and dark respiration (via dissolved oxygen) as well as changes in pH of the three different assemblages: a healthy and mature forest, a restored forest, and a degraded assemblage (Figure 2a). All studied localities were shallower than 1 m depth.

### Incubation setup

The optimal experimental design for the benthic incubations would have been to conduct the experiments simultaneously across the three studied assemblages. However, this was not feasible due to logistic limitations, as each fieldwork day required five people, and the distance between the two bays was >50 km. As a result, in situ benthic incubations of the three different assemblages were conducted over consecutive days with consistent and stable weather conditions over the fieldwork campaign (April 2022). We measured the in situ water temperature (°C), salinity, above‐surface irradiance (μmol photons · m^−2^ · s^−1^), pH (on the total scale), and dissolved oxygen (mg O_2_ · L^−^^1^) every day prior to the incubations to account for any sudden change in water and atmospheric conditions that may have influenced the incubation outcomes. Dissolved oxygen and pH were measured with temperature‐corrected probes (Hach sensION+ DO6 and Hach sensION+ pH 1 – 5052 T, respectively), which show the measurement of the environmental variable and current temperature. The oxygen and pH probes were calibrated to 100% oxygen every day before the fieldwork and with 7 and 10 pH calibration solutions, respectively. Salinity was measured with a salinometer (ATAGO, ES‐421), and above‐surface irradiance was measured with a LI‐COR light sensor (LI 1500 with LI‐193SA spherical quantum sensor). Above‐surface irradiance was transformed to estimated underwater irradiance using the Beer–Lambert equation (Gordon, 1989) and with the light attenuation coefficient (K) set at 1.42, as observed in a coastal lagoon also in Menorca (Obrador & Petrus, 2008). Since macroalgal production reaches its maximum saturation at ~250 μmol · photons · m^−2^ · s^−1^ (Ballesteros & Sant, 2022), production outcomes were not influenced by weather conditions given that estimated underwater irradiance was always above 800 μmol photons · m^−2^ · s^−1^ throughout the incubation periods.

The incubation design consisted of a dome‐shaped chamber (approximately 17.5 L, 42 cm in diameter) with a rigid frame measuring 4 cm at the base (Figure 2b,c). We used transparent and opaque, black methacrylate chambers for the light and dark conditions, respectively (Manipulats de Plàstics Garro S. L., Ripollet, Barcelona). The light condition allowed us to estimate community net primary production (NPP), whereas the dark condition accounted for community dark respiration. Each chamber was connected to an automated external pump that flushed water at a rate of 240 L · h^−1^, ensuring the complete mixing of the water enclosed in the chamber and the disruption of the diffusion boundary layer that may accumulate on the seaweed surface during incubation (Hurd, 2000). The pump was powered by a 12 V waterproof battery operated by a float switch. During the incubations, each chamber was carefully sealed using Lycra bags (175 × 25 cm) loaded with surrounding sand and placed around the chamber frame. The incubation setup was followed and adapted from Peleg et al. (2020). The sealing of the incubation system was verified by injecting fluorescein dye into the chambers and examining the perimeters for leaks (e.g., Edwards et al., 2020; Miller et al., 2009).

For each assemblage (i.e., degraded, mature forest, and restored forest), we randomly deployed six replicate incubation chambers. The light incubations were always first conducted around midday, guaranteeing the period of highest light availability. After that, the dark chambers were placed in the same position as the light ones, ensuring that both conditions incubated the same surface area. Prior to the incubations, the chambers were placed over the communities for 30 min to allow all the organisms to acclimatize to the new light conditions, which was especially important prior to the dark incubations (Florez‐Sarasa et al., 2012). Immediately afterward, we carefully lifted the chamber to fully replace the water inside it before putting it back and completely sealing the chamber with the sandbags. We ran incubations for 1 h and 30 min (average time 1 h and 32 ± 6 min), as a balance among the time used in Peleg et al. (2020), the required field time for the different steps and data collection, and the impact of long incubation periods on primary production outcomes as reported by Olivé et al. (2016). At the beginning and end of each incubation, 200 mL of seawater from the chambers was sampled through a tube and a sampling port using 100‐mL polyethylene syringes. All the syringes were rinsed three times with the surrounding water before each sampling. Sampled water was carefully transferred into a 45‐mL falcon tube, avoiding any bubble formation, to immediately measure dissolved oxygen and pH (Figure 2b).

### Community data sampling

For each chamber, a representative area (20 × 20 cm) of the community was removed with a hammer and chisel after the incubations. Samples were fixed in a 4% seawater‐formalin solution for further processing in the laboratory. Macroalgal sorting was performed with the naked eye, and all individuals measuring more than 0.5 cm were identified to species or genera level. Macroinvertebrates were sorted into broad taxonomic categories (i.e., Polychaeta, Gastropoda, Amphipoda, etc.) in three different chambers per assemblage, to estimate the proportion of photosynthetic biomass versus heterotrophic biomass of each assemblage. Biomass was obtained as dry weight (g) after 48 h of drying at 60°C (e.g., Ballesteros & Sant, 2022; Galobart et al., 2023; Sant et al., 2017).

### Primary production and dark respiration estimations

Community NPP was estimated as the difference between the final and initial dissolved oxygen concentrations in the light incubations, while community dark respiration (R) was estimated from the dissolved oxygen concentration of the dark incubations. Both rates were normalized by the chamber area, volume, and incubation time according to the formula:
(1)

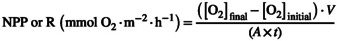

where [O_2_] is the dissolved oxygen concentration (mmol O_2_ · L^−1^), *V* is the volume of the chamber (~17.5 L), *A* is the area enclosed in the chamber (42 cm in diameter; ~0.1384 m^2^), and *t* is the duration of each incubation (h). Community gross primary production (GPP) was then calculated according to the following formula:
(2)

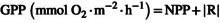




### Statistical analyses

A type I one‐way analysis of variance (ANOVA) was conducted to analyze the effect of assemblage (three levels: “degraded,” “forest,” and “restored forest”) on the dry biomass of macroalgae and macroinvertebrates and the number of individuals of macroinvertebrates. Then, we used a 2D nonmetric multidimensional scaling (nMDS) ordination analysis (Bray‐Curtis dissimilarities) to investigate patterns of variation in species composition (stress value <0.1). We used dry biomass for macroalgal species and ln(*x* + 1) transformed abundance data for macroinvertebrates, to weigh down the contribution of very abundant groups on the overall ordination of samples. We checked differences in oxygen metabolic rates (NPP, R, GPP) with the same ANOVA design, and we tested differences in pH between the start and end of incubations with a one‐way repeated measures ANOVA with time (two levels: “initial” and “final”) as a fixed factor. For the different ANOVAs, the normality of residuals was assessed by looking at quantile–quantile plots and Shapiro–Wilk tests, while homogeneity of variance was tested by means of the “residuals versus fitted values” plot and Levene's tests, respectively. Whenever significant differences were observed, these were investigated by running a Tukey pairwise comparison. All analyses were conducted in R version 4.0.2 (R Core Team, 2018).

## RESULTS

The seawater temperature before the incubations was 20.08 ± 1.06°C, salinity was 37, dissolved oxygen was 7.12 ± 0.25 mg O_2_ · L^−1^, and pH was 8.22 ± 0.01 (Table S1). Estimated underwater irradiance stayed at between 800 and 950 μmol photons · m^−2^ · s^−1^ during midday light incubations.

**FIGURE 3:**
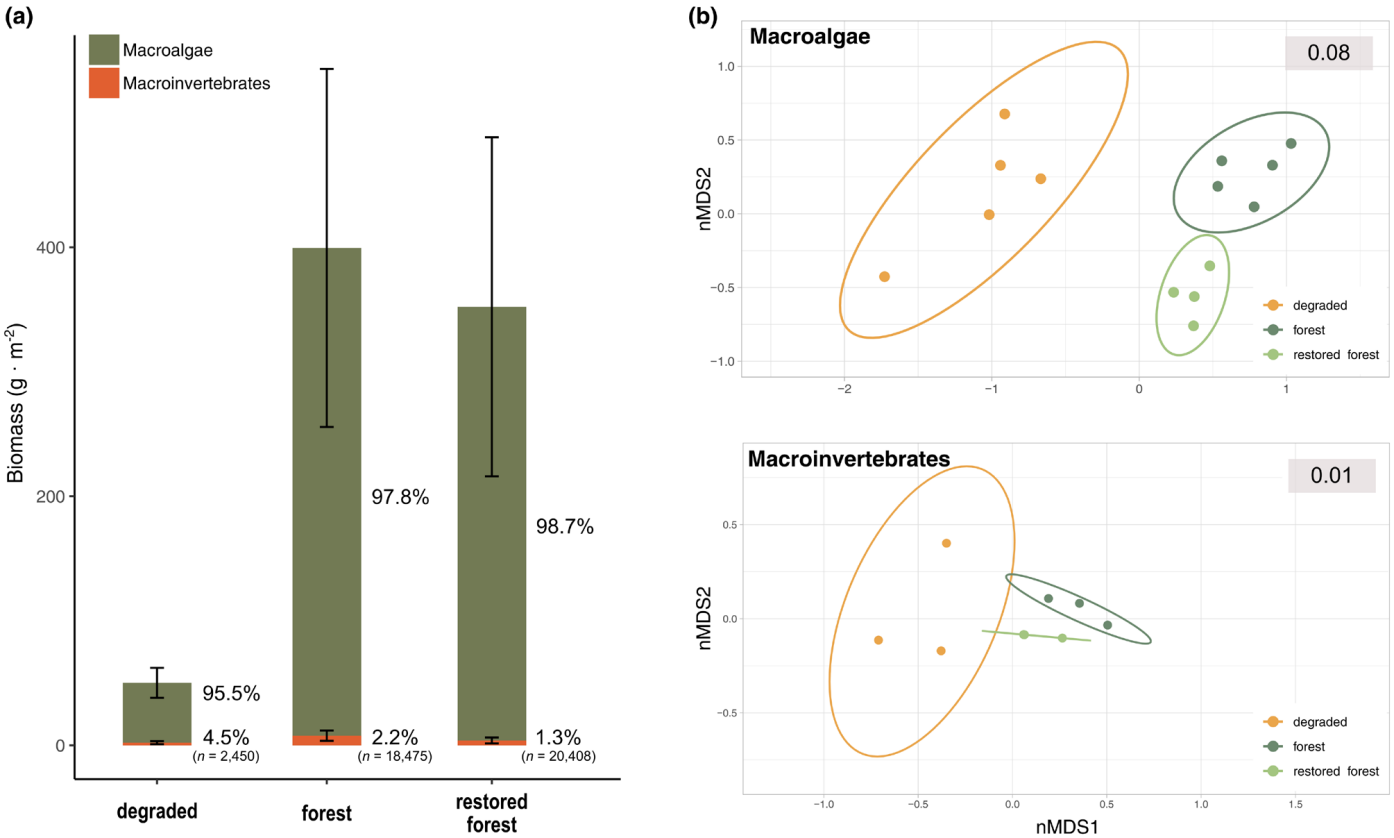
(a) Biomass of photosynthetic species (25 macroalgae and one seagrass) and macroinvertebrates, as dry weight (g · m^−2^). Values represent the percentage of photosynthetic vs. heterotrophic biomass within each assemblage. *n* corresponds to the number of total macroinvertebrate individuals found in each assemblage (ind · m^−2^). (b) 2D nonmetric multidimensional scaling ordination of macroalgae and macroinvertebrates, using dry weight for macroalgae and ln(*x* + 1) transformed abundance for macroinvertebrates. Ellipses are 95% confidence intervals around the centroids of each assemblage.

Macroalgal biomass significantly varied in the different assemblages (ANOVA, *F* (2, 11) = 18.58, *p* = 0.0003, Tables S2 and S3). Specifically, the results showed minimum values in the degraded assemblage, with 48.2 ± 11.2 g · m^−2^ of biomass (Table S2). Macroalgal biomass was similar and significantly higher in the mature and restored forests at 359 ± 116 and 366.8 ± 115 g · m^−2^, respectively (Table S3). Macroinvertebrate biomass did not differ among assemblages (ANOVA, *F* (2, 6) = 3.097, *p* = 0.119, Tables S3 and S4) and represented less than 5% of total biomass (i.e., macroalgae and macroinvertebrates) in all three studied assemblages (Figure 3a). However, the number of macroinvertebrate individuals differed among assemblages (ANOVA, *F* (2, 6) = 23.85, *p* = 0.0014, Table S3), being higher in the mature and restored forests compared to the degraded assemblage, with 20,408 ± 10,342 ind · m^−2^ in the mature forest, 18,475 ± 9497 ind · m^−2^ in the restored forest, and 2450 ± 254 ind · m^−2^ in the degraded assemblage (Figure 3a; Table S4). The ordination plots of both macroalgae and macroinvertebrates showed that the species compositions of the mature and restored forests were more similar than that of the degraded one, displaying closer positions in the ordination space (Figure 3b). Moreover, samples from the degraded assemblage showed greater variability, characterized by a wider distance between them and a larger ellipse compared to the other assemblages (Figure 3b). The difference in photosynthetic biomass was mainly due to the higher abundance of *Gongolaria barbata* in the forested assemblages. In the healthy and mature forest, we also observed other abundant species accompanying *G. barbata*, such as *Dictyota* sp., *Anadyomene stellata*, and *Cymodocea nodosa*. Similarly, *Dictyota* sp. and *C. nodosa*, together with other species, such as *Halopteris scoparia* and *Sphacelaria* sp., were observed in the restored forest. In the degraded assemblage, the most dominant species was *Padina pavonica*, together with *Lophosiphonia* sp. and *Dictyota* sp. (Table S2).

**FIGURE 4:**
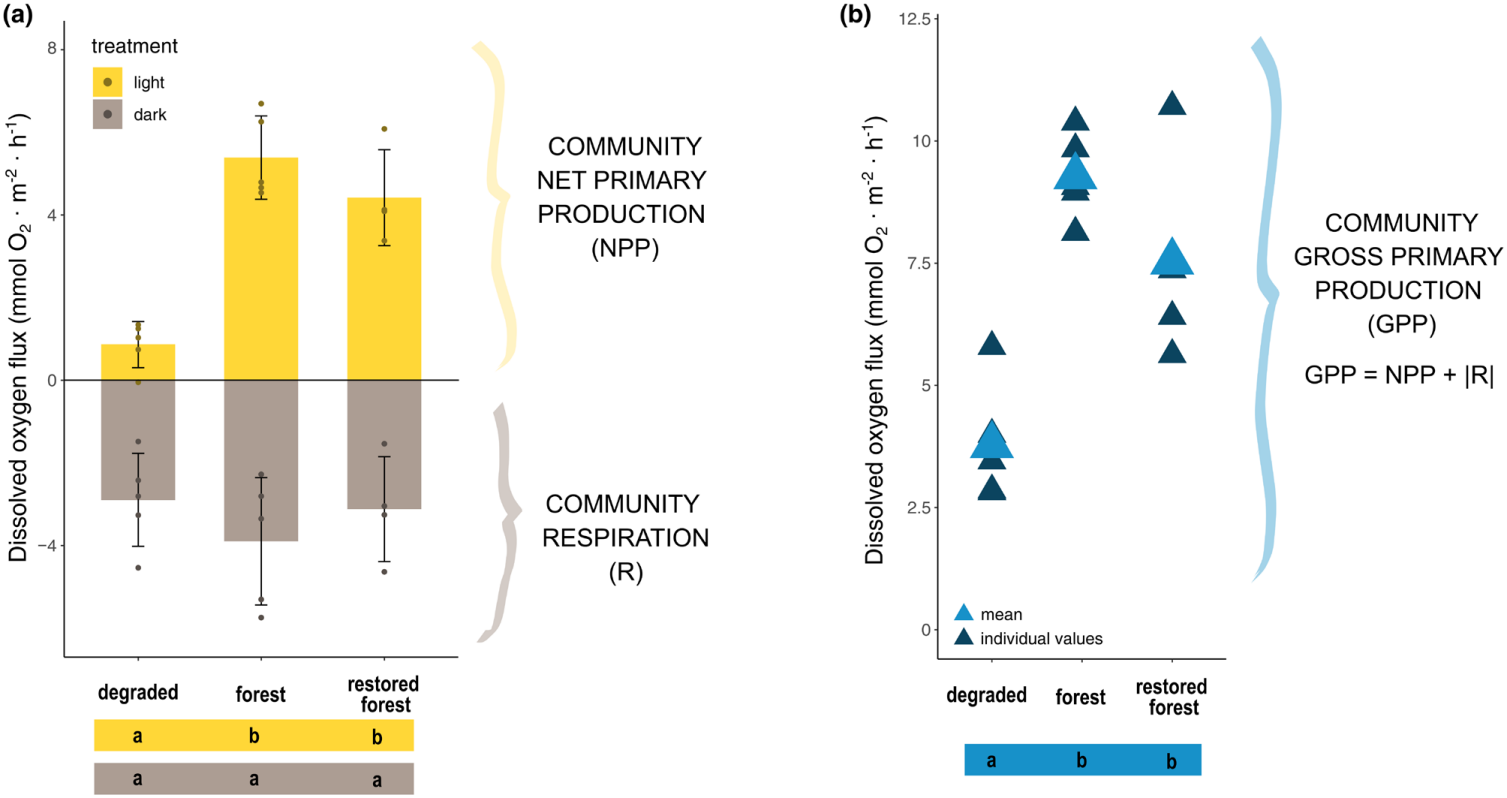
Community metabolic rates in the three studied assemblages (degraded, forest, and restored forest). (a) Community net primary production (NPP) and dark respiration (R) estimated via oxygen evolution in light and dark incubations, respectively. Error bars correspond to standard deviation (*SD*). (b) Community gross primary production (GPP), calculated from NPP and R values. Dissimilar letters under plots indicate significant differences (Tukey post hoc pairwise test within 95% confidence intervals).

Community NPP differed across assemblages (ANOVA, *F* (2, 11) = 32.8, *p* < 0.0001, Figure 4a; Table S5). The NPP of the degraded assemblage was always significantly lower than those in the mature and restored forests, with NPP values of 0.86 ± 0.6 mmol O_2_ · m^−2^ · h^−1^ (Figure 4a; Table S5). The NPP values were similar in the mature and restored forests, measuring 5.38 ± 1.01 and 4.41 ± 1.16 mmol O_2_ · m^−2^ · h^−1^, respectively (Table S5). These results showed that the NPP was 5.1 times higher in the restored assemblage than in the degraded one, and 5.7 times higher than the average values of both the mature and restored forests. Community dark respiration (R) showed no variation among assemblages (ANOVA, *F* (2, 11) = 0.77, *p* = 0.487, Figure 4a; Table S5), with an average of −3.32 ± 1.30 mmol O_2_ · m^−2^ · h^−1^. As a result, GPP also differed across assemblages (ANOVA, *F* (2, 11) = 17.95, *p* = 0.0003, Table S5). In particular, the GPP of the degraded assemblage was 3.78 ± 1.22 mmol O_2_ · m^−2^ · h^−1^, significantly lower than in the mature and restored forests, at 9.28 ± 0.87 and 7.53 ± 2.24 mmol O_2_ · m^−2^ · h^−1^, respectively (Figure 4b; Table S5). In this case, the GPP of the restored forest was two times higher than in the degraded assemblage and 2.2 times higher than the average of the restored and mature forests.

**FIGURE 5:**
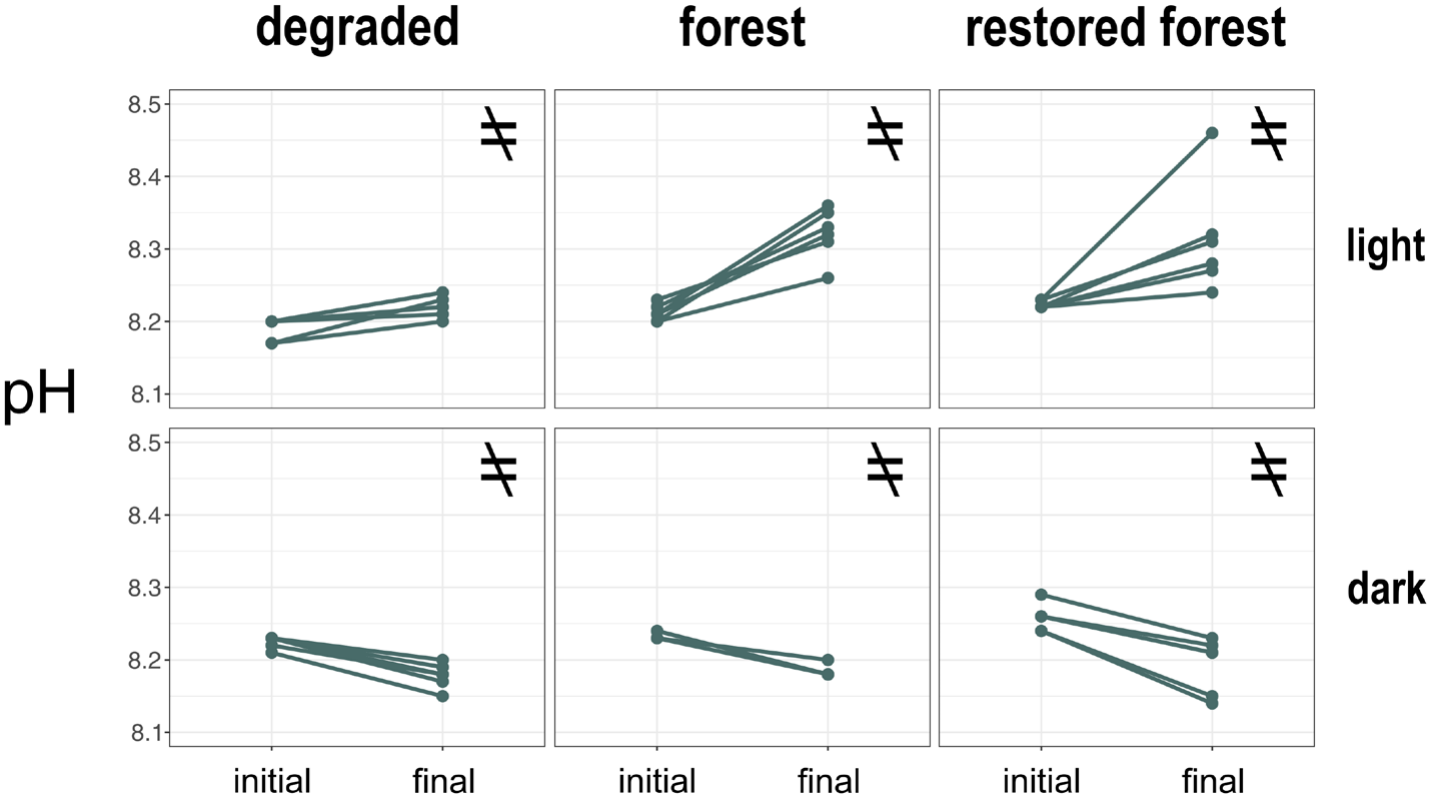
Initial and final measures of pH during light (upper row) and dark (lower row) incubations at the three studied assemblages. One‐way repeated measures ANOVA was used to test for differences between initial and final measures. An unequal symbol indicates significant differences, whereas an equal symbol indicates no differences.

We observed differences between the initial and final pH in both light and dark incubations in all assemblages (repeated measures ANOVA, light degraded: *F* (1, 5) = 21.74, *p* = 0.0055; light forest: *F* (1, 5) = 55, *p* = 0.0007; light‐restored forest: *F* (1, 5) = 8.93, *p* = 0.0305; dark degraded: *F* (1, 5) = 89.09, *p* = 0.0002; dark forest: *F* (1, 3) = 50, *p* = 0.0058; dark‐restored forest: *F* (1, 5) = 42.97, *p* = 0.0012, Figure 5; Table S6). The pH significantly increased in the light incubations, specifically in the restored and mature forest assemblages, which showed an increase from 8.22 ± 0.01 to 8.31 ± 0.08 and from 8.21 ± 0.01 to 8.32 ± 0.03, respectively (Figure 5). In the degraded assemblage, the pH change was less pronounced ranging from 8.19 ± 0.02 to 8.22 ± 0.02 during incubation. The increase in pH was three and 3.7 times greater in the restored and mature forests, respectively, than in the degraded assemblage. Contrastingly, pH significantly decreased in the dark incubations, although it showed a similar pattern in all assemblages (Figure 5; Table S6). A detailed summary of the field data and the estimated oxygen fluxes for each incubation is provided in Table S7.

## DISCUSSION

In this study, we have shown the recovery of the oxygen and pH fluxes in a 10‐year Mediterranean marine restored forest, which achieved rates comparable to those in a healthy and mature forest habitat. The average net oxygen production occurring during light periods in the restored forest assemblage was 5.1 times higher than that of the degraded one. Similarly, the pH increase was three times greater in the restored forest than in the degraded assemblage. The macroalgal biomass and the abundance of macroinvertebrate species were also significantly higher in the restored forest, with a 7.6‐fold increase in macroalgal biomass and an eight‐fold increase in macroinvertebrate abundance. Overall, our results show the potential of active restoration actions to recover ecosystem processes and functions, such as oxygen production and habitat provisioning.

Considering the oxygen cycle, all three assemblages showed a positive NPP flux, indicating that all of them produced more oxygen than they consumed and acted as net autotrophs during the incubation periods. However, the average NPP value of the mature and restored forests was 5.7 times higher than that of the degraded assemblage, with a mean net oxygen production of 4.95 ± 1.1 mmol O_2_ · m^−2^ · h^−1^. Many studies quantifying seaweed production using benthic chambers have focused on single species (e.g., Rodgers et al., 2015; White & Davoult, 2022), and others have performed the incubations over different time scales (e.g., days, in Edwards et al., 2020), making it challenging to compare (and convert) our data with similar systems. Although we acknowledge the limited evidence of comparable in situ community measurements, our results fall in the lower range of observations, varying from negative or close to zero NPP estimates in foliose macroalgal communities recorded in the Atlantic (Miller et al., 2009) to values of ~17 mmol O_2_ · m^−2^ · h^−1^ reported for a Mediterranean marine macroalgal forest also dominated by Fucalean species (Peleg et al., 2020). The elevated NPP standardized by area obtained in Peleg et al. (2020) may be attributed to the abundance of producers in their study because the photosynthetic biomass in the macroalgal habitat they examined was considerably higher. In fact, the biomass of the photosynthetic species showed a six‐fold increase in the restored forest compared to the degraded assemblage, displaying similar values to the mature forest. This suggests that the higher primary production obtained in the mature and restored forests was also primarily driven by higher macroalgal biomass.

Community dark respiration rates did not vary across the three studied assemblages. Several authors have observed that macroalgal species presenting a thin and foliose thallus exhibit higher rates of dark respiration related to their biomass than more structurally complex species (Enríquez et al., 1995; Sant & Ballesteros, 2020). Two of the most abundant species in the degraded assemblage were *Padina pavonica* and *Dictyota* sp., both characterized by a foliose morphology (blades) that contrasts with the tree‐like shape of the Fucalean species *Gongolaria barbata* dominating the forested assemblages. No differences were observed between macroinvertebrate biomass, and its percentage was relatively small compared to photosynthetic biomass (<5%). However, the degraded assemblage showed a slightly higher proportion of macroinvertebrates compared with the mature and restored forests (4.5% vs. 2.2% and 1.3%, respectively). The identities of the macroalgal species in the degraded assemblage and the macroinvertebrate proportion may have influenced the dark respiration outcomes in this study. Although macroinvertebrate biomass was similar in all three assemblages, the numbers of individuals in the restored and mature forests were significantly higher compared to the degraded assemblage. This result may indicate a recovery of habitat provision following restoration, potentially due to increased habitat complexity provided by *G. barbata* and the associated macroalgal community (Galobart et al., 2023). The few studies that have assessed faunal recovery after macroalgal restoration generally observed changes in species composition compared to degraded assemblages, although not always complete resemblance to reference assemblages (e.g. Bianchelli et al., 2023; Christie et al., 2024; Marzinelli et al., 2016). The recovery of macroinvertebrate diversity linked to macroalgal restoration can be a complex, long‐term process that may affect different species in varying ways. A more detailed classification and identification of species could help in understanding the recovery patterns of specific macroinvertebrates associated with certain algal species but would require a comprehensive biodiversity assessment.

During the light incubations, there was a greater pH increase in the mature and restored assemblages than in the degraded ones, which is in accordance with the oxygen fluxes identified. The uptake of inorganic carbon via photosynthesis raises water pH levels (Frankignoulle, 1994; Gattuso et al., 1995), and our results, therefore, indicate that aerobic metabolism was dominant during the light incubations and suggest inorganic carbon assimilation. These results showcase that the daytime dynamics of the seawater carbonate system in the restored forest have changed and have become more like those observed in the mature and healthy forest. However, the overall functioning of dissolved inorganic carbon cycle in the studied assemblages remains unclear because inorganic carbon is also involved in other complex community processes such as calcification/carbonate dissolution (e.g., Barrón et al., 2006; Kalokora et al., 2020) and anaerobic processes derived from microbial activity (e.g. Forja et al., 2004). Additional data on other variables informing about the carbonate system (e.g., total alkalinity) are required to completely understand the dissolved inorganic carbon dynamics of the studied assemblages.

Different methods to estimate macroalgal production (e.g., biomass standing stock and accumulation, in situ incubations, laboratory experiments, and aquatic eddy covariance, among others) can yield different metabolic outcomes (e.g., Brinkhuis, 1977; Tait & Schiel, 2010; White et al., 2021), so direct comparisons between available data should be cautiously interpreted. Moreover, daily and seasonal primary production measurements are required if the goal is for data to be included in global NPP estimations (e.g., Pessarrodona et al., 2022), and it must be borne in mind that spatial variations and local conditions greatly influence these measurements (Edwards et al., 2020). Therefore, the strength of our study relies on the estimation of oxygen and pH changes with the same method and under similar conditions in contrasting habitats, allowing the comparison of ecosystem processes along a structural complexity gradient (degraded, mature, and restored assemblages).

Considering the values obtained in the mature and restored assemblages dwelling in different localities (Bay of Fornells and Bay of Maó), our results may suggest differences in species composition and ecosystem processes between healthy, well‐preserved habitats and their degraded counterparts. Although only one degraded assemblage was incubated, it exhibited a reduction in its oxygen production and potential carbon assimilation, underlining the significance of conservation initiatives to prevent the loss of ecosystem functions and services in natural and healthy ecosystems. Our work also provides empirical evidence that once a single structural species has been successfully restored in the early stages and species diversity has been recovered, recovering important ecosystem processes can yield positive results. The recovery of primary production was most likely linked to the recovery of biomass; thus, the functional ecosystem recovery could not have been achieved in a short time period, since at least 6 years were needed to recover the density and size structure of *Gongolaria barbata* populations in the area (Verdura et al., 2018). This highlights the necessity of long‐term monitoring in marine forest restoration actions, in particular when dealing with long‐lived species and when restoration techniques involve the use of recruitment enhancement, where adults are not directly provided. This is the only available long‐term restoration action in the Mediterranean; as such, we cannot ascertain that the findings of this study are generalizable to other macroalgal restoration efforts, evidencing, therefore, a need for further data on long‐term success indicators for restoration assessments encompassing different geographical regions and environmental conditions. In light of the required time to recover the processes, the costs of macroalgal restoration efforts (see Eger et al., 2022 for a review), and the uncertainties and challenges to predict successful outcomes, we strongly support precautionary conservation initiatives to protect and preserve the existing natural ecosystems and advocate that conservation and restoration efforts should work as complementary approaches.

## AUTHOR CONTRIBUTIONS

**Cristina Galobart:** Conceptualization (supporting); formal analysis (lead); investigation (equal); writing – original draft (lead); writing – review and editing (equal). **Cèlia Sitjà:** Investigation (equal); writing – review and editing (equal). **Sònia de Caralt:** Investigation (equal); writing – review and editing (equal). **Jorge Santamaría:** Investigation (equal); writing – review and editing (equal). **Alba Vergés:** Investigation (equal); writing – review and editing (equal). **Jordi Boada:** Investigation (equal); writing – review and editing (equal). **Emma Cebrian:** Conceptualization (lead); funding acquisition (lead); investigation (equal); writing – original draft (supporting); writing – review and editing (equal).

## Supporting information


**Table S1.** Daily in situ water temperature, dissolved oxygen (DO), pH (on the total scale), and salinity before the light incubations, measured around midday (12 p.m.).

**Table S2.** Identified macrophyte species (25 macroalgae and one seagrass) and corresponding biomass. Values are mean ± SD dry weight in grams per square meter. Only individuals measuring more than 0.5 cm were identified and weighed.

**Table S3.** One‐way ANOVA summary of (a) macroalgae and (b) macroinvertebrates biomass, (c) number of macroinvertebrate individuals, and (d), (e) their pairwise comparisons. The *p*‐values of the post hoc test were adjusted with the Tukey method. The number of individuals in the macroinvertebrate abundance matrix was ln (x+1) transformed previously to analysis. The asterisk (*) indicates a significant *p*‐value.

**Table S4.** Identified macroinvertebrate groups. Upper values are mean ± SD number of individuals per square meter and lower values are mean ± SD dry weight in grams. Gastropoda and Bivalvia weights were measured after removing the shells of the individuals.

**Table S5.** (a, c, d) One‐way ANOVA summary of oxygen metabolic rates (NPP, R, and GPP) across assemblages. (b, e) Pairwise comparisons. The *p*‐values of the post hoc test were adjusted with the Tukey method. The asterisk (*) indicates a significant *p*‐value.

**Table S6.** One‐way repeated measures ANOVA summary of pH data from light and dark incubations and across assemblages. The asterisk (*) indicates a significant *p*‐value.

**Table S7.** Detailed summary table of incubation data and estimated oxygen fluxes.

## Data Availability

The data and R code supporting the findings of this study are available in the Github digital repository (https://github.com/cgalobart/Macroalgal‐restoration‐metabolic‐fluxes).

## References

[jpy13520-bib-0001] Abdullah, M. I. , & Fredriksen, S. (2004). Production, respiration and exudation of dissolved organic matter by the kelp *Laminaria hyperborea* along the west coast of Norway. Journal of the Marine Biological Association of the United Kingdom, 84, 887–894. 10.1017/S002531540401015Xh

[jpy13520-bib-0002] Airoldi, L. , & Beck, M. W. (2007). Loss, status and trends for coastal marine habitats of Europe. In R. N. Gibson , R. J. A. Atkinson , & J. D. M. Gordon (Eds.), Oceanography and marine biology: An annual review (pp. 345–405). Taylor & Francis. 10.1201/9781420050943.ch7

[jpy13520-bib-0003] Aronson, J. , & Alexander, S. (2013). Ecosystem restoration is now a global priority: Time to roll up our sleeves. Restoration Ecology, 21, 293–296. 10.1111/rec.12011

[jpy13520-bib-0004] Ballesteros, E. (1992). *Els vegetals i la zonació litoral: especies, comunitats i factors queinflueixen en la seva distribució* [PhD thesis]. Arxius Secció Ciències, 101, Institut d'Estudis Catalans.

[jpy13520-bib-0005] Ballesteros, E. , & Sant, N. (2022). Homogeneity of photosynthetic features in canopy‐forming macroalgae ofthe order Fucales from shallow and sheltered environments. Cryptogamie Algologie, 43(6), 107–115. 10.5252/cryptogamie-algologie2022v43a6

[jpy13520-bib-0006] Barrón, C. , Duarte, C. M. , Frankignoulle, M. , & Borges, A. V. (2006). Organic carbon metabolism and carbonate dynamics in a Mediterranean seagrass (*Posidonia oceanica*) meadow. Estuaries and Coasts, 29, 417–426. 10.1007/BF02784990

[jpy13520-bib-0007] Bennett, S. , Wernberg, T. , Connell, S. D. , Hobday, A. J. , Johnson, C. R. , & Poloczanska, E. S. (2016). The “Great Southern Reef”: Social, ecological and economic value of Australia's neglected kelp forests. Marine and Freshwater Research, 67, 47–56. 10.1071/MF15232

[jpy13520-bib-0008] Bianchelli, S. , Fraschetti, S. , Martini, F. , Lo Martire, M. , Nepote, E. , Ippoliti, D. , & Danovaro, R. (2023). Macroalgal forest restoration: The effect of the foundation species. Frontiers in Marine Science, 10, 1213184. 10.3389/fmars.2023.1213184

[jpy13520-bib-0009] Bordeyne, F. , Migné, A. , Plus, M. , & Davolut, D. (2020). Modelling the annual primary production of an intertidal brown algal community based on *in situ* measurements. Marine Ecology Progress Series, 656, 95–107. 10.3354/meps13450

[jpy13520-bib-0010] Brinkhuis, B. H. (1977). Comparisons of salt‐marsh fucoid production estimated from three different indices. Journal of Phycology, 13, 328–335. 10.1111/j.1529-8817.1977.tb02936.x

[jpy13520-bib-0011] Cebrian, E. , Tamburello, L. , Verdura, J. , Guarnieri, G. , Medrano, A. , Linares, C. , Hereu, B. , Garrabou, J. , Cerrano, C. , Galobart, C. , & Fraschetti, S. (2021). A roadmap for the restoration of Mediterranean macroalgal forests. Frontiers in Marine Science, 8, e709219. 10.3389/fmars.2021.709219

[jpy13520-bib-0012] Cheminée, A. , Sala, E. , Pastor, J. , Bodilis, P. , Thiriet, P. , Mangialajo, L. , Cottalorda, J. M. , & Francour, P. (2013). Nursery value of *Cystoseira* forests for Mediterranean rocky reef fishes. Journal of Experimental Marine Biology and Ecology, 442, 70–79. 10.1016/j.jembe.2013.02.003

[jpy13520-bib-0013] Christie, H. , Moy, F. E. , Fagerli, C. W. , Rinde, E. , Strand, M. , Tveiten, L. A. , & Strand, H. K. (2024). Successful large‐scale and long‐term kelp forest restoration by culling sea urchins with quicklime and supported by crab predation. Marine Biology, 171, 211. 10.1007/s00227-024-04540-0

[jpy13520-bib-0014] Christie, H. , Norderhaug, K. M. , & Fredriksen, S. (2009). Macrophytes as habitat for fauna. Marine Ecology Progress Series, 396, 221–233. 10.3354/meps08351

[jpy13520-bib-0015] Costanza, R. , d'Arge, R. , de, R. , Farber, S. , Grasso, M. , Hannon, B. , Limburg, K. , Naeem, S. , O'Neill, R. , Paruelo, J. , Raskin, R. , Sutton, P. , & van, M. (1997). The value of the world's ecosystem services and natural capital. Nature, 387, 253–260. 10.1038/387253a0

[jpy13520-bib-0016] de Caralt, S. , Verdura, J. , Santamaría, J. , Vergés, A. , & Cebrian, E. (2023). Importance of life history traits for vulnerability to climate change: Implications for macroalgal restoration. Frontiers in Marine Science, 10, 1248629. 10.3389/fmars.2023.1248629

[jpy13520-bib-0017] Díez, I. , Santolaria, A. , Secilla, A. , & Gorostiaga, J. M. (2009). Recovery stages over long‐term monitoring of the intertidal vegetation in the “Abra de Bilbao” area and on the adjacent coast (N. Spain). European Journal of Phycology, 44, 1–14. 10.1080/09670260802158642

[jpy13520-bib-0018] Duarte, C. M. (2017). Reviews and syntheses: Hidden forests, the role of vegetated coastal habitats in the ocean carbon budget. Biogeosciences, 14, 301–310. 10.5194/bg-14-301-2017

[jpy13520-bib-0019] Earp, H. S. , Smale, D. , Pérez‐Matus, A. , Gouraguine, A. , Shaw, P. W. , & Moore, P. J. (2022). A quantitative synthesis of approaches, biases, successes, and failures in marine forest restoration, with considerations for future work. Aquatic Conservation: Marine and Freshwater Ecosystems, 32, 1717–1731. 10.1002/aqc.3880

[jpy13520-bib-0020] Edwards, M. , Konar, B. , Kim, J. H. , Gabara, S. , Sullaway, G. , McHugh, T. , Spector, M. , & Small, S. (2020). Marine deforestation leads to widespread loss of ecosystem function. PLoS ONE, 15, e0226173. 10.1371/journal.pone.0226173 32130220 PMC7055868

[jpy13520-bib-0021] Eger, A. M. , Marzinelli, E. M. , Beas‐Luna, R. , Blain, C. O. , Blamey, L. K. , Byrnes, J. E. K. , Carnell, P. , Choi, C. , Hessing‐Lewis, M. , Kim, K. , Kumagai, N. , Lorda, J. , Moore, P. , Nakamura, Y. , Pérez‐Matus, A. , Pontier, O. , Smale, D. , Steinberg, P. , & Vergés, A. (2023). The value of ecosystem services in global marine kelp forests. Nature Communications, 14, 1894. 10.1038/s41467-023-37385-0 PMC10113392 37072389

[jpy13520-bib-0022] Eger, A. M. , Marzinelli, E. M. , Christie, H. , Fagerli, C. W. , Fujita, D. , Gonzalez, A. P. , Hong, S. , Kim, J. , Lee, L. , McHugh, T. , Nishihara, G. , Tatsumi, M. , Steinberg, P. , & Vergés, A. (2022). Global kelp forest restoration: Past lessons, present status, and future directions. Biological Reviews, 97, 1449–1475. 10.1111/brv.12850 35255531 PMC9543053

[jpy13520-bib-0023] Enríquez, S. , Duarte, C. M. , & Sand Jensen, K. (1995). Patterns in the photosynthetic metabolism of Mediterranean macrophytes. Marine Ecology Progress Series, 119, 243–252. 10.3354/meps119243

[jpy13520-bib-0024] European Commission: Directorate‐General for the Environment . (2013). Interpretation manual of European Union habitats (vers. EUR28). European Commission.

[jpy13520-bib-0025] European Commission: Directorate‐General for Environment . (2022). Nature restoration law: For people, climate, and planet. Publications Office of the European Union. 10.2779/86148

[jpy13520-bib-0026] Filbee‐Dexter, K. , & Wernberg, T. (2018). Rise of turfs: A new battlefront for globally declining kelp forests. Bioscience, 68, 64–76. 10.1093/biosci/bix147

[jpy13520-bib-0027] Florez‐Sarasa, I. , Araújo, W. L. , Wallström, S. V. , Rasmusson, A. G. , Fernie, A. R. , & Ribas‐Carbo, M. (2012). Light‐responsive metabolite and transcript levels are maintained following a dark‐adaptation period in leaves of *Arabidopsis thaliana* . New Phytologist, 195, 136–148. 10.1111/j.1469-8137.2012.04153.x 22548389

[jpy13520-bib-0028] Forja, J. M. , Ortega, T. , Del Valls, T. A. , & Gómez‐Parra, A. (2004). Benthic fluxes of inorganic carbon in shallow coastal ecosystems of the Iberian Peninsula. Marine Chemistry, 85, 141–156. 10.1016/j.marchem.2003.09.007

[jpy13520-bib-0029] Frankignoulle, M. (1994). A complete set of buffer factors for acid/base CO_2_ system in seawater. Journal of Marine Systems, 5, 111–118. 10.1016/0924-7963(94)90026-4

[jpy13520-bib-0030] Fredriksen, S. , Filbee‐Dexter, K. , Norderhaug, K. M. , Steen, H. , Bodvin, T. , Coleman, M. A. , Moy, F. , & Wernberg, T. (2020). Green gravel: A novel restoration tool to combat kelp forest decline. Scientific Reports, 10, 3983. 10.1038/s41598-020-60553-x 32132550 PMC7055217

[jpy13520-bib-0031] Galobart, C. , Ballesteros, E. , Golo, R. , & Cebrian, E. (2023). Addressing marine restoration success: Evidence of species and functional diversity recovery in a ten‐year restored macroalgal forest. Frontiers in Marine Science, 10, e1176655. 10.3389/fmars.2023.1176655

[jpy13520-bib-0032] Gann, G. D. , McDonald, T. , Walder, B. , Aronson, J. , Nelson, C. R. , Jonson, J. , & Dixon, K. W. (2019). International principles and standards for the practice of ecological restoration. Second Edition. Restoration Ecology, 27, S1–S46. 10.1111/rec.13035

[jpy13520-bib-0033] Gattuso, J. P. , Pichon, M. , & Frankignoulle, M. (1995). Biological control of air‐sea CO_2_ fluxes: Effect of photosynthetic and calcifying marine organisms and ecosystems. Marine Ecology Progress Series, 129, 307–312. 10.3354/meps129307

[jpy13520-bib-0034] Golléty, C. , Migné, A. , & Davoult, D. (2008). Benthic metabolism on a sheltered rocky shore: Role of the canopy in the carbon budget. Journal of Phycology, 44, 1146–1153. 10.1111/j.1529-8817.2008.00569.x 27041711

[jpy13520-bib-0035] Gordon, H. R. (1989). Can the Lambert‐Beer law be applied to the diffuse attenuation coefficient of ocean water? Limnology and Oceanography, 34, 1389–1409. 10.4319/lo.1989.34.8.1389

[jpy13520-bib-0036] Govern de les Illes Balears . (2007). Implementació de la Directiva Marc de l’Aigua a les Illes Balears: Avaluació de la qualitat ambiental de les masses d’aigua costaneres utilitzant les macroalgues i els invertebrats bentònics coma bioindicadors [Implementation of the Water Framework Directive in the Balearic Islands: Assessment of the environmental quality of coastal water bodies using macroalgae and benthic invertebrates ad bioindicators]. Conselleria de Medi Ambient.

[jpy13520-bib-0037] Gran, A. , Movilla, J. , Ballesteros, E. , Sales, M. , Bolado, I. , Galobart, C. , & Cefalì, M. E. (2022). Assessing the expansion and success of a restored population of *Gongolaria barbata* (Stackhouse) Kuntze (Fucales, Phaeophyceae) using high‐precision positioning tools and size distribution frequencies. Mediterranean Marine Science, 23, 907–916. 10.12681/mms.30500

[jpy13520-bib-0038] Heimann, M. , & Reichstein, M. (2008). Terrestrial ecosystem carbon dynamics and climate feedbacks. Nature, 451, 289–292. 10.1038/nature06591 18202646

[jpy13520-bib-0039] Helsinki Commission . (2013). Red list of Baltic Sea underwater biotopes, habitats and biotope complexes. (Baltic Sea Environmental Proceedings No. 138). Baltic Marine Environmental Protection Commission ‐ HELCOM.

[jpy13520-bib-0040] Hoyo, X. (1981). El Port de Maó: un ecosistema de gran interès ecològic i didàctic [The Port of Maó: An ecosystem of great ecological and educational interest]. Maina, 3, 32–37.

[jpy13520-bib-0041] Hurd, C. L. (2000). Water motion, marine macroalgal physiology, and production. Journal of Phycology, 36, 453–472. 10.1046/j.1529-8817.2000.99139.x 29544017

[jpy13520-bib-0042] Iveša, L. , Djakovac, T. , & Devescovi, M. (2016). Long‐term fluctuations in *Cystoseira* populations along the west Istrian Coast (Croatia) related to eutrophication patterns in the northern Adriatic Sea. Marine Pollution Bulletin, 106, 162–173. 10.1016/j.marpolbul.2016.03.010 26975612

[jpy13520-bib-0043] Kalokora, O. J. , Buriyo, A. S. , Asplund, M. E. , Gullström, M. , Mtolera, M. S. P. , & Björk, M. (2020). An experimental assessment of algal calcification as a potential source of atmospheric CO_2_ . PLoS ONE, 15, e0231971. 10.1371/journal.pone.0231971 32348324 PMC7190104

[jpy13520-bib-0044] Layton, C. , Cameron, M. J. , Shelamoff, V. , Tatsumi, M. , Wright, J. T. , & Johnson, C. R. (2021). A successful method of transplanting adult *Ecklonia radiata* kelp, and relevance to other habitat‐forming macroalgae. Restoration Ecology, 29, e13412. 10.1111/rec.13412

[jpy13520-bib-0045] Leith, H. , & Whittaker, R. (Eds.). (1975). Primary productivity of the biosphere. Springer‐Verlag. 10.1007/978-3-642-80913-2

[jpy13520-bib-0046] Ling, S. D. , Ibbott, S. , & Sanderson, J. C. (2010). Recovery of canopy‐forming macroalgae following removal of the enigmatic grazing sea urchin *Heliocidaris erythrogramma* . Journal of Experimental Marine Biology and Ecology, 395, 135–146. 10.1016/j.jembe.2010.08.027

[jpy13520-bib-0047] Mann, K. H. (1973). Seaweeds: Their productivity and strategy for growth. Science, 182, 975–981. 10.1126/science.182.4116.975 17833778

[jpy13520-bib-0048] Mariani, S. , Cefalì, M. E. , Chappuis, E. , Terradas, M. , Pinedo, S. , Torras, X. , Jordana, E. , Medrano, A. , Verdura, J. , & Ballesteros, E. (2019). Past and present of Fucales from shallow and sheltered shores in Catalonia. Regional Studies in Marine Science, 32, 100824. 10.1016/j.rsma.2019.100824

[jpy13520-bib-0049] Marzinelli, E. M. , Leong, M. R. , Campbell, A. H. , Steinberg, P. D. , & Vergés, A. (2016). Does restoration of a habitat‐forming seaweed restore associated faunal diversity? Restoration Ecology, 24, 81–90. 10.1111/rec.12292

[jpy13520-bib-0050] Michałek, M. , & Kruk‐Dowgiałło, L. (2016). Criteria for the conservation status assessment of the marine habitats. Case study: Habitat 1160 Large, shallow inlets and bays. Bulletin of the Maritime Institute in Gdańsk, 31(1), 1–6. 10.5604/12307424.1196263

[jpy13520-bib-0051] Miller, R. J. , Reed, D. C. , & Brzezinski, M. A. (2009). Community structure and productivity of subtidal turf and foliose algal assemblages. Marine Ecology Progress Series, 388, 1–11. 10.3354/meps08131

[jpy13520-bib-0052] Minguito‐Frutos, M. , Adams, M. P. , Alcoverro, T. , Vilas, M. P. , Alonso, D. , Mayol, E. , Bernardeu‐Esteller, J. , Marín‐Guirao, L. , Ruiz, J. M. , & Boada, J. (2023). Quantifying the role of photoacclimation and self‐facilitation for seagrass resilience to light deprivation. Frontiers in Plant Science, 14, 1186538. 10.3389/fpls.2023.1186538 37546272 PMC10401047

[jpy13520-bib-0053] Molinari Novoa, E. A. , & Guiry, M. D. (2020). Reinstatement of the genera *Gongolaria* Boehmer and *Ericaria* Stackhouse (Sargassaceae, Phaeophyceae). Notulae Algarum, 172, 1–10.

[jpy13520-bib-0054] Moy, F. E. , & Christie, H. (2012). Large‐scale shift from sugar kelps (*Saccharina latissima*) to ephemeral algae along the south and west coast of Norway. Marine Biology Research, 8, 309–321. 10.1080/17451000.2011.637561

[jpy13520-bib-0055] Mtwana Nordlund, L. , Koch, E. W. , Barbier, E. B. , & Creed, J. C. (2016). Seagrass ecosystem services and their variability across genera and geographical regions. PLoS ONE, 11, e0163091. 10.1371/journal.pone.0163091 27732600 PMC5061329

[jpy13520-bib-0056] Obrador, B. , & Petrus, J. L. (2008). Light regime and components of turbidity in a Mediterranean coastal lagoon. Estuarine, Coastal and Shelf Science, 77, 123–133. 10.1016/j.ecss.2007.09.008

[jpy13520-bib-0057] Olivé, I. , Silva, J. , Costa, M. M. , & Santos, R. (2016). Estimating seagrass community metabolism using benthic chambers: The effect of incubation time. Estuaries and Coasts, 39, 138–144. 10.1007/s12237-015-9973-z

[jpy13520-bib-0058] Orellana, S. , Hernández, M. , & Sansón, M. (2019). Diversity of *Cystoseira sensu lato* (Fucales, Phaeophyceae) in the eastern Atlantic and Mediterranean based on morphological and DNA evidence, including *Carpodesmia* gen. emend. and *Treptacantha* gen. emend. European Journal of Phycology, 54, 447–465. 10.1080/09670262.2019.1590862

[jpy13520-bib-0059] Orfanidis, S. , Rindi, F. , Cebrian, E. , Fraschetti, S. , Nasto, I. , Taskin, E. , & Danovaro, R. (2021). Effects of natural and anthropogenic stressors on Fucalean brown seaweeds across different spatial scales in the Mediterranean Sea. Frontiers in Marine Science, 8, e658417. 10.3389/fmars.2021.658417

[jpy13520-bib-0060] Peleg, O. , Guy‐Haim, T. , Yeruham, E. , Silverman, J. , & Rilov, G. (2020). Tropicalization may invert trophic state and carbon budget of shallow temperate rocky reefs. Journal of Ecology, 108, 844–854. 10.1111/1365-2745.13329

[jpy13520-bib-0061] Perring, M. P. , Standish, R. J. , Price, J. N. , Craig, M. D. , Erickson, T. E. , Ruthrof, K. X. , Whiteley, A. S. , Valentine, L. E. , & Hobbs, R. J. (2015). Advances in restoration ecology: Rising to the challenges of the coming decades. Ecosphere, 6, 131. 10.1890/ES15-00121.1

[jpy13520-bib-0062] Pessarrodona, A. , Assis, J. , Filbee‐Dexter, K. , Burrows, M. T. , Gattuso, J. P. , Duarte, C. M. , Krause‐Jensen, D. , Moore, P. , Smale, D. , & Wernberg, T. (2022). Global seaweed productivity. Science Advances, 8, 1–10. 10.1126/sciadv.abn2465 PMC9473579 36103524

[jpy13520-bib-0063] Piazzi, L. , Bonaviri, C. , Castelli, A. , Ceccherelli, G. , Costa, G. , Curini‐Galletti, M. , Langeneck, J. , Manconi, R. , Montefalcone, M. , Pipitone, C. , Rosso, A. , & Pinna, S. (2018). Biodiversity in canopy‐forming algae: Structure and spatial variability of the Mediterranean *Cystoseira* assemblages. Estuarine, Coastal and Shelf Science, 207, 132–141. 10.1016/j.ecss.2018.04.001

[jpy13520-bib-0064] Pinedo, S. , Zabala, M. , & Ballesteros, E. (2013). Long‐term changes in sublittoral macroalgal assemblages related to water quality improvement. Botanica Marina, 56, 461–469. 10.1515/bot-2013-0018

[jpy13520-bib-0065] R Core Team . (2018). R: A language and environment for statistial computing. https://www.r‐project.org/

[jpy13520-bib-0066] Reed, D. C. , Schroeter, S. C. , & Raimondi, P. T. (2004). Spore supply and habitat availability as sources of recruitment limitation in the giant kelp *Macrocystis pyrifera* (Phaeophyceae). Journal of Phycology, 40, 275–284. 10.1046/j.1529-8817.2004.03119.x

[jpy13520-bib-0067] Ribera, G. , Coloreu, M. , Rodríguez‐Prieto, C. , & Ballesteros, E. (1997). Phytobenthic assemblages of Addaia Bay (Menorca, western Mediterranean): Composition and distribution. Botanica Marina, 40, 523–532. 10.1515/botm.1997.40.1-6.523

[jpy13520-bib-0068] Rocha, J. , Yletyinen, J. , Biggs, R. , Blenckner, T. , & Peterson, G. (2015). Marine regime shifts: Drivers and impacts on ecosystems services. Philosophical Transactions of the Royal Society B, 370, 20130273. 10.1098/rstb.2013.0273

[jpy13520-bib-0069] Rodgers, K. L. , Rees, T. A. V. , & Shears, N. T. (2015). A novel system for measuring in situ rates of photosynthesis and respiration of kelp. Marine Ecology Progress Series, 528, 101–115. 10.3354/meps11273

[jpy13520-bib-0070] Sales, M. , & Ballesteros, E. (2009). Shallow *Cystoseira* (Fucales: Ochrophyta) assemblages thriving in sheltered areas from Menorca (NW Mediterranean): Relationships with environmental factors and anthropogenic pressures. Estuarine, Coastal and Shelf Science, 84, 476–482. 10.1016/j.ecss.2009.07.013

[jpy13520-bib-0071] Sales, M. , & Ballesteros, E. (2010). Long‐term comparison of algal assemblages dominated by *Cystoseira crinita* (Fucales, Heterokontophyta) from Cap Corse (Corsica, North Western Mediterranean). European Journal of Phycology, 45, 404–412. 10.1080/09670262.2010.498585

[jpy13520-bib-0072] Sales, M. , Cebrian, E. , Tomas, F. , & Ballesteros, E. (2011). Pollution impacts and recovery potential in three species of the genus *Cystoseira* (Fucales, Heterokontophyta). Estuarine, Coastal and Shelf Science, 92, 347–357. 10.1016/j.ecss.2011.01.008

[jpy13520-bib-0073] Sant, N. , & Ballesteros, E. (2020). Photosynthetic activity of macroalgae along a bathymetric gradient: Interspecific and seasonal variability. Scientia Marina, 84, 7–16. 10.3989/scimar.04995.06A

[jpy13520-bib-0074] Sant, N. , Chappuis, E. , Rodríguez‐Prieto, C. , Real, M. , & Ballesteros, E. (2017). Cost‐benefit of three different methods for studying Mediterranean rocky benthic assemblages. Scientia Marina, 81, 129–138. 10.3989/scimar.04463.04A

[jpy13520-bib-0075] Smith, C. J. , Verdura, J. , Papadopoulou, N. , Fraschetti, S. , Cebrian, E. , Fabbrizzi, E. , Monserrat, M. , Drake, M. , Bianchelli, S. , Danovaro, R. , Malak, D. , Ballesteros, E. , Benjumea, T. , Boissery, P. , D’Ambrosio, P. , Galobart, C. , Javel, F. , Laurent, D. , Orfanidis, S. , & Mangialajo, L. (2023). A decision‐support framework for the restoration of *Cystoseira sensu lato* forests. Frontiers in Marine Science, 10, 1159262. 10.3389/fmars.2023.1159262

[jpy13520-bib-0076] Suding, K. , Higgs, E. , Palmer, M. , Callicott, J. B. , Anderson, C. B. , Baker, M. , Gutrich, J. , Hondula, K. , LaFevor, M. , Larson, B. , Randall, A. , Ruhl, J. , & Schwartz, K. (2015). Committing to ecological restoration. Science, 348, 638–640. 10.1126/science.aaa4216 25953995

[jpy13520-bib-0077] Tait, L. W. , & Schiel, D. R. (2010). Primary productivity of intertidal macroalgal assemblages: Comparison of laboratory and in situ photorespirometry. Marine Ecology Progress Series, 416, 115–125. 10.3354/meps08781

[jpy13520-bib-0078] Teagle, H. , Hawkins, S. J. , Moore, P. J. , & Smale, D. A. (2017). The role of kelp species as biogenic habitat formers in coastal marine ecosystems. Journal of Experimental Marine Biology and Ecology, 492, 81–98. 10.1016/j.jembe.2017.01.017

[jpy13520-bib-0079] Thayer, G. W. , Wolfe, D. A. , & Willimas, R. W. (1975). The impact of man on seagrass systems. American Scientist, 63, 228–296.

[jpy13520-bib-0080] Verdura, J. , Sales, M. , Ballesteros, E. , Cefalì, M. E. , & Cebrian, E. (2018). Restoration of a canopy‐forming alga based on recruitment enhancement: Methods and long‐term success assessment. Frontiers in Plant Science, 9, 1832. 10.3389/fpls.2018.01832 30619405 PMC6295557

[jpy13520-bib-0081] Vergés, A. , McCosker, E. , Mayer‐Pinto, M. , Coleman, M. A. , Wernberg, T. , Ainsworth, T. , & Steinberg, P. D. (2019). Tropicalisation of temperate reefs: Implications for ecosystem functions and management actions. Functional Ecology, 33, 1000–1013. 10.1111/1365-2435.13310

[jpy13520-bib-0082] Vergés, A. , Tomas, F. , Cebrian, E. , Ballesteros, E. , Kizilkaya, Z. , Dendrinos, P. , Karamanlidis, A. , Spiegel, D. , & Sala, E. (2014). Tropical rabbit fish and the deforestation of a warming temperate sea. Journal of Ecology, 102, 1518–1527. 10.1111/1365-2745.12324

[jpy13520-bib-0083] Wernberg, T. , & Filbee‐Dexter, K. (2019). Missing the forest for the trees. Marine Ecology Progress Series, 612, 209–215. 10.3354/meps12867

[jpy13520-bib-0084] White, L. , & Davoult, D. (2022). Photosynthetic capacity of co‐occurring kelp species revealed by in situ measurements. Marine Ecology Progress Series, 697, 31–43. 10.3354/meps14152

[jpy13520-bib-0085] White, L. , Loisel, S. , Sevin, L. , & Davoult, D. (2021). In situ estimates of kelp forest productivity in macro‐tidal environments. Limnology and Oceanography, 66, 4227–4239. 10.1002/lno.11955

