# Supplementary material for: Oxygen and pH fluxes in shallow bay habitats: Evaluating the effectiveness of a macroalgal forest restoration
Source: J Phycol. 2024 Nov 18;61(1):20–33. doi: 10.1111/jpy.13520 (PMC11914953; doi:10.1111/jpy.13520)
Supplement: Supplementary file 1 — Table S1. Daily in situ water temperature, dissolved oxygen (DO), pH (on the total scale), and salinity before the light incubations, measured around midday (12 p.m.). [file JPY-61-20-s001.docx]

**Supporting Information**

**Table S1.** Daily *in situ* water temperature, dissolved oxygen (DO), pH (on the total scale) and salinity before the light incubations, measured around midday (12 pm).

|  | **replicate** | **temp (ºC)** | **DO (mg O_2_ L^-1^)** | **pH** | **salinity (ppm)** |
| --- | --- | --- | --- | --- | --- |
| **degraded** | 1 | 19.10 | 7.17 | 8.24 |  |
|  | 2 | 19.40 | 6.86 | 8.22 |  |
|  | 3 | 20.10 | 7.26 | 8.25 |  |
|  | **average** | **19.53** | **7.10** | **8.24** | **37** |
|  | **SD** | **0.51** | **0.21** | **0.02** | **-** |
| **forest** | 1 | 19 | 7.25 | 8.23 |  |
|  | 2 | 19.7 | 7.04 | 8.21 |  |
|  | 3 | 19.3 | 6.76 | 8.22 |  |
|  | **average** | **19.33** | **7.02** | **8.22** | **37** |
|  | **SD** | **0.35** | **0.25** | **0.01** | **-** |
| **restored forest** | 1 | 20.9 | 7.05 | 8.22 |  |
|  | 2 | 21.2 | 7.09 | 8.22 |  |
|  | 3 | 22 | 7.61 | 8.21 |  |
|  | **average** | **21.37** | **7.25** | **8.22** | **37** |
|  | **SD** | **0.57** | **0.31** | **0.01** | **-** |
| **total** | **average** | **20.08** | **7.12** | **8.22** | **37** |
|  | **SD** | **1.06** | **0.25** | **0.01** | **-** |
