# Supplementary material for: Oxygen and pH fluxes in shallow bay habitats: Evaluating the effectiveness of a macroalgal forest restoration
Source: J Phycol. 2024 Nov 18;61(1):20–33. doi: 10.1111/jpy.13520 (PMC11914953; doi:10.1111/jpy.13520)
Supplement: Supplementary file 2 — Table S2. Identified macrophyte species (25 macroalgae and one seagrass) and corresponding biomass. Values are mean ± SD dry weight in grams per square meter. Only individuals measuring more than 0.5 cm were identified and weighed. [file JPY-61-20-s004.docx]

**Supporting Information**

**Table S2.** Identified macrophyte species (25 macroalgae and 1 seagrass) and corresponding biomass. Values are mean ± SD of dry weight in grams per square meter. Only individuals measuring more than 0.5 cm were identified and weighed.

|  | **degraded** | **forest** | **restored forest** |
| --- | --- | --- | --- |
|  | (n = 5) | (n = 5) | (n = 4) |
| *Acetabularia acetabulum* | 0.04 ± 0.1 | 0.1 ± 0.1 | - |
| *Alsidium helminthochorton* | 1.2 ± 2.6 | - | - |
| *Amphiroa rigida* | 3.9 ± 8.1 | - | - |
| *Anadyomene stellata* | - | 19.3 ± 12.8 | - |
| *Caulerpa cylindracea* | - | - | 0.6 ± 0.5 |
| *Caulerpa prolifera* | - | 14.8 ± 19.5 | 0.3 ± 0.6 |
| *Ceramium* sp. | 1.1 ± 2.4 | - | 0.1 ± 0.2 |
| *Chordaceae* non identified | 0.01 ± 0.01 | 0.01 ± 0.01 | 1.4 ± 1.6 |
| *Cladophora prolifera* | - | - | 0.3 ± 0.4 |
| *Cladophora* sp. | 1.1 ± 2.5 | 0.5 ± 0.4 | 28.9 ± 19.1 |
| *Cymodocea nodosa* | 0.2 ± 0.5 | 8.6 ± 10.6 | 22.4 ± 31.2 |
| *Cystoseira* s.l. branches | - | - | 6.7 ± 4.9 |
| *Cystoseira foeniculacea* f. *tenuiramosa* | - | - | 2.5 ± 5 |
| *Cystoseira pustulata* | - | - | 6.7 ± 10.3 |
| *Dasycladus vermicularis* | - | 1.6 ± 3.4 | - |
| *Dictyota* sp. | 6 ± 7.5 | 41.9 ± 13 | 40.7 ± 22.3 |
| *Gongolaria barbata* | - | 249.8 ± 119.8 | 163.1 ± 61.1 |
| *Gongolaria montagnei* | - | 7.5 ± 14.6 | - |
| *Halopteris scoparia* | - | - | 49.8 ± 28.6 |
| *Jania virgata* | 1.2 ± 2.6 | - | 9.5 ± 16.3 |
| *Lophosiphonia* sp. 1 | 7.6 ± 6.9 | - | - |
| *Lophosiphonia* sp. 2 | 0.4 ± 0.9 | - | - |
| *Padina pavonica* | 25.4 ± 11.7 | 1.8 ± 2.3 | 6.2 ± 4.2 |
| *Rytiphlaea tinctoria* | - | 0.8 ± 1 | - |
| *Sphacelaria* sp. | - | 4.6 ± 5.7 | 27.4 ± 30.5 |
| *Stilophora tenella* | - | 3.6 ± 3 | 0.3 ± 0.3 |
| *Valonia utricularis* | - | 0.1 ± 0.1 | - |
| **Total (g of dry weight · m^-2^)** | **48.1 ± 11.2** | **359 ± 116.1** | **366.8 ± 115** |
