# Supplementary material for: Oxygen and pH fluxes in shallow bay habitats: Evaluating the effectiveness of a macroalgal forest restoration
Source: J Phycol. 2024 Nov 18;61(1):20–33. doi: 10.1111/jpy.13520 (PMC11914953; doi:10.1111/jpy.13520)
Supplement: Supplementary file 3 — Table S3. One‐way ANOVA summary of (a) macroalgae and (b) macroinvertebrates biomass, (c) number of macroinvertebrate individuals, and (d), (e) their pairwise comparisons. The p‐values of the post hoc test were adjusted with the Tukey method. The number of individuals in the macroinvertebrate abundance matrix was ln (x+1) transformed previously to analysis. The asterisk (*) indicates a significant p‐value. [file JPY-61-20-s006.docx]

**Supporting Information**

**Table S3.** One-way ANOVA summary of (A) macroalgae and (B) macroinvertebrates biomass), (C) number of macroinvertebrate individuals, and (D), (E) their pairwise comparisons. P-values of the post hoc test were adjusted with the Tukey method. The number of individuals in the macroinvertebrate abundance matrix was ln+1 transformed previously to analysis. The asterisk (*) indicates a significant p-value.

| **(A) biomass macroalgae** |  |  |  |  |  |
| --- | --- | --- | --- | --- | --- |
| **one-way ANOVA** | **Df** | **Sum Sq** | **Mean Sq** | **F value** | **Pr (>F)** |
| assemblage | 2 | 317720 | 158860 | 18.58 | 0.0003* |
| residuals | 11 | 94060 | 8551 |  |  |
|  |  |  |  |  |  |
| **(B) biomass macroinvertebrates** |  |  |  |  |  |
| **one-way ANOVA** | **Df** | **Sum Sq** | **Mean Sq** | **F value** | **Pr (>F)** |
| assemblage | 2 | 49 | 25 | 3.097 | 0.119 |
| residuals | 6 | 48 | 8 |  |  |

| **(C) number of individuals macroinvertebrates** | |  |  |  |  |  |
| --- | --- | --- | --- | --- | --- | --- |
| **one-way ANOVA** | | **Df** | **Sum Sq** | **Mean Sq** | **F value** | **Pr (>F)** |
| assemblage | | 2 | 7.939 | 3.970 | 23.85 | 0.0014* |
| residuals | | 6 | 0.999 | 0.166 |  |  |
|  |  |  |  |  |  |  |
| **(D) biomass macroalgae** | |  |  |  |  |  |
| **comparison** | | **diff** | **lwr** | **upr** | **p adj** |  |
| forest - degraded | | 310.840 | 152.88 | 468.80 | 0.0007* |  |
| restored forest - degraded | | 318.690 | 151.16 | 486.23 | 0.0009* |  |
| restored forest - forest | | 7.854 | -159.68 | 175.39 | 0.991 |  |

| **(E) number of individuals macroinvertebrates** |  |  |  |  |  |
| --- | --- | --- | --- | --- | --- |
| **comparison** | **diff** | **lwr** | **upr** | **p adj** |  |
| forest - degraded | 1.9378 | 0.9156 | 2.9599 | 0.0027* |  |
| restored forest - degraded | 2.0428 | 1.0207 | 3.0650 | 0.0021* |  |
| restored forest - forest | 0.1050 | -0.9171 | 1.1272 | 0.947 |  |
