# Supplementary material for: Oxygen and pH fluxes in shallow bay habitats: Evaluating the effectiveness of a macroalgal forest restoration
Source: J Phycol. 2024 Nov 18;61(1):20–33. doi: 10.1111/jpy.13520 (PMC11914953; doi:10.1111/jpy.13520)
Supplement: Supplementary file 4 — Table S4. Identified macroinvertebrate groups. Upper values are mean ± SD number of individuals per square meter and lower values are mean ± SD dry weight in grams. Gastropoda and Bivalvia weights were measured after removing the shells of the individuals. [file JPY-61-20-s003.docx]

**Supporting Information**

**Table S4.** Identified macroinvertebrate groups. Upper values are mean ± SD of number of individuals per square meter and lower values are mean ± SD of dry weight in grams. Gastropoda and Bivalvia weight was measured after removing the shell of the individuals.

|  | **degraded** | **forest** | **restored forest** |
| --- | --- | --- | --- |
|  | (n = 3) | (n = 3) | (n = 3) |
| Polychaeta | 892 ± 315 | 5,683 ± 5,257 | 3,225 ± 1,645 |
|  | (0.226 ± 0.107) | (0.672 ± 0.611) | (0.254 ± 0.154) |
| Amphipoda | 500 ± 125 | 3,150 ± 1,988 | 9,250 ± 6,261 |
|  | (0.063 ± 0.045) | (0.216 ± 0.153) | (0.513 ± 0.34) |
| Isopoda | 233 ± 128 | 508 ± 539 | 2,300 ± 548 |
|  | (0.009 ± 0.004) | (0.264 ± 0.371) | (0.208 ± 0.263) |
| Cumacea | 17 ± 14 | 1,533 ± 838 | 1,292 ± 989 |
|  | (0.003) | (0.014 ± 0.001) | (0.017 ± 0.016) |
| Tanaidacea | 142 ± 181 | 958 ± 742 | 517 ± 409 |
|  | (0.003) | (0.021 ± 0.013) | (0.001 ± 0.002) |
| Pycnogonida | 8 ± 14 | 58 ± 58 | 75 ± 43 |
|  | (0.001 ± 0.002) | (0.004 ± 0.003) | (0.002 ± 0.001) |
| Echinodermata (Ophiurida) | 8 ± 14 | 292 ± 484 | 433 ± 253 |
|  | (0.001 ± 0.002) | (0.021 ± 0.02) | (0.128 ± 0.126) |
| Gastropoda | 600 ± 175 | 3,267 ± 2,327 | 1,192 ± 853 |
|  | (1.573 ± 1.049) | (3.672 ± 1.614) | (2.238 ± 1.829) |
| Bivalvia | 33 ± 38 | 300 ± 195 | 267 ± 194 |
|  | (0.176 ± 0.295) | (1.462 ± 1.994) | (0.069 ± 0.069) |
| Polyplacophora | 17 ± 29 | 25 ± 0 | - |
|  | (0.066 ± 0.114) | (0.003 ± 0.003) |  |
| Chironomidae | - | 1,892 ± 1,121 | 1,300 ± 763 |
|  |  | (0.081 ± 0.044) | (0.036 ± 0.025) |
| Acari | - | 642 ± 577 | 500 ± 517 |
|  |  | (0.003) | (0.003) |
| Ascidiacea | - | 108 ± 72 | 50 ± 87 |
|  |  | (0.794 ± 0.63) | (0.414 ± 0.716) |
| Porifera | - | 33 ± 58 | 8 ± 14 |
|  |  | (0.398 ± 0.689) | (0.002 ± 0.003) |
| Heterobranchia | - | 25 ± 25 | - |
|  |  | (0.089 ± 0.153) |  |
| **Total (ind · m^-2^)** | 2,450 ± 254 | 18,475 ± 9,497 | 20,408 ± 10,342 |
| **Total (g of dry weight · m^-2^)** | (2.121 ± 1.210) | (7.715 ± 4.069) | (3.884 ± 2.398) |
