# Supplementary material for: Oxygen and pH fluxes in shallow bay habitats: Evaluating the effectiveness of a macroalgal forest restoration
Source: J Phycol. 2024 Nov 18;61(1):20–33. doi: 10.1111/jpy.13520 (PMC11914953; doi:10.1111/jpy.13520)
Supplement: Supplementary file 5 — Table S5. (a, c, d) One‐way ANOVA summary of oxygen metabolic rates (NPP, R, and GPP) across assemblages. (b, e) Pairwise comparisons. The p‐values of the post hoc test were adjusted with the Tukey method. The asterisk (*) indicates a significant p‐value. [file JPY-61-20-s005.docx]

**Supporting Information**

**Table S5. A), C), D)** One-way ANOVA summary of oxygen metabolic rates (NPP, R, GPP) across assemblages. **B), E)** Pairwise comparisons. P-values of the post hoc test were adjusted with the Tukey method. The asterisk (*) indicates a significant p-value.

| **(A) NPP** |  |  |  |  |  |
| --- | --- | --- | --- | --- | --- |
| **one-way ANOVA** | **Df** | **Sum Sq** | **Mean Sq** | **F value** | **Pr (>F)** |
| assemblage | 2 | 56.040 | 28.018 | 32.8 | <0.0001* |
| residuals | 11 | 9.390 | 0.854 |  |  |
|  |  |  |  |  |  |
| **(B) NPP** |  |  |  |  |  |
| **comparison** | **diff** | **lwr** | **upr** | **p adj** |  |
| forest - degraded | 4.528 | 2.949 | 6.107 | <0.0001* |  |
| restored forest - degraded | 3.557 | 1.883 | 5.231 | 0.0003* |  |
| restored forest - forest | -0.971 | -2.645 | 0.703 | 0.2999 |  |
|  |  |  |  |  |  |
|  |  |  |  |  |  |
|  |  |  |  |  |  |
| **(C) R** |  |  |  |  |  |
| **one-way ANOVA** | **Df** | **Sum Sq** | **Mean Sq** | **F value** | **Pr (>F)** |
| assemblage | 2 | 2.704 | 1.352 | 0.77 | 0.487 |
| residuals | 11 | 19.319 | 1.756 |  |  |
|  |  |  |  |  |  |
|  |  |  |  |  |  |
|  |  |  |  |  |  |
| **(D) GPP** |  |  |  |  |  |
| **one-way ANOVA** | **Df** | **Sum Sq** | **Mean Sq** | **F value** | **Pr (>F)** |
| assemblage | 2 | 78.56 | 39.28 | 17.95 | 0.0003* |
| residuals | 11 | 24.08 | 2.19 |  |  |
|  |  |  |  |  |  |
| **(E) GPP** |  |  |  |  |  |
| **comparison** | **diff** | **lwr** | **upr** | **p adj** |  |
| forest - degraded | 5.503 | 2.976 | 8.030 | 0.0003* |  |
| restored forest - degraded | 3.751 | 1.071 | 6.432 | 0.0079* |  |
| restored forest - forest | -1.752 | -4.432 | 0.929 | 0.2260 |  |
