# Supplementary material for: Oxygen and pH fluxes in shallow bay habitats: Evaluating the effectiveness of a macroalgal forest restoration
Source: J Phycol. 2024 Nov 18;61(1):20–33. doi: 10.1111/jpy.13520 (PMC11914953; doi:10.1111/jpy.13520)
Supplement: Supplementary file 6 — Table S6. One‐way repeated measures ANOVA summary of pH data from light and dark incubations and across assemblages. The asterisk (*) indicates a significant p‐value. [file JPY-61-20-s007.docx]

**Supporting Information**

**Table S6:** One-way repeated measures ANOVA summary of pH data from light and dark incubations and across assemblages. The asterisk (*) indicates a significant p-value.

| **pH** |  |  |  |  |  |  |  |
| --- | --- | --- | --- | --- | --- | --- | --- |
|  |  |  | **df** | **sum Sq** | **mean Sq** | **F value** | **Pr (>F)** |
| light | degraded | time | 1 | 0.0033 | 0.0033 | 21.74 | 0.0055* |
|  |  | residuals | 5 | 0.0008 | 0.0001 |  |  |
|  | forest | time | 1 | 0.0363 | 0.0363 | 55 | 0.0007* |
|  |  | residuals | 5 | 0.0033 | 0.0007 |  |  |
|  | restored forest | time | 1 | 0.0243 | 0.0243 | 8.93 | 0.0305* |
|  |  | residuals | 5 | 0.0136 | 0.0027 |  |  |
| dark | degraded | time | 1 | 0.0065 | 0.0065 | 89.09 | 0.0002* |
|  |  | residuals | 5 | 0.0004 | 0.0001 |  |  |
|  | forest | time | 1 | 5x10^-3^ | 5x10^-3^ | 50 | 0.0058* |
|  |  | residuals | 3 | 5x10^-4^ | 1x10^-4^ |  |  |
|  | restored forest | time | 1 | 0.0127 | 0.0127 | 42.97 | 0.0012* |
|  |  | residuals | 5 | 0.0015 | 0.0003 |  |  |
|  |  |  |  |  |  |  |  |
