# Supplementary material for: Oxygen and pH fluxes in shallow bay habitats: Evaluating the effectiveness of a macroalgal forest restoration
Source: J Phycol. 2024 Nov 18;61(1):20–33. doi: 10.1111/jpy.13520 (PMC11914953; doi:10.1111/jpy.13520)
Supplement: Supplementary file 7 — Table S7. Detailed summary table of incubation data and estimated oxygen fluxes. [file JPY-61-20-s002.docx]

**Supporting Information**

**Table S7:** Detailed summary table of incubation data and estimated oxygen fluxes.

|  |  |  | **DO initial** (mg O_2_ L^-1^) | **DO final** (mg O_2_ L^-1^**)** | **pH initial** | **pH final** | **Incubation time** (min) | **Dry biomass macroalgae** (g m^-2^) | **Dry biomass macroinvertebrates** (g m^-2^) | **DO flux**  (mmol O_2_ m^-2^ h^-1^) |
| --- | --- | --- | --- | --- | --- | --- | --- | --- | --- | --- |
| **degraded** | **light** | **1** | 7.16 | 7.64 | 8.17 | 8.2 | 91 | 60.5 | 1.015 | 1.25 |
|  |  | **2** | - | - | 8.17 | 8.23 | 93 | - | - | - |
|  |  | **3** | 7.26 | 7.78 | 8.2 | 8.21 | 92 | 35.5 | - | 1.34 |
|  |  | **4** | 7.36 | 7.76 | 8.2 | 8.22 | 92 | 37.0 | 3.141 | 1.03 |
|  |  | **5** | 7.40 | 7.38 | 8.2 | 8.24 | 95 | 54.5 | - | -0.05 |
|  |  | **6** | 7.34 | 7.64 | 8.2 | 8.24 | 97 | 53.3 | 1.933 | 0.74 |
|  | **dark** | **1** | 7.33 | 5.59 | 8.22 | 8.18 | 91 | 60.5 | 1.015 | -4.54 |
|  |  | **2** | - | - | 8.23 | 8.17 | 91 | - | - | - |
|  |  | **3** | 7.56 | 6.98 | 8.23 | 8.2 | 93 | 35.5 | - | -1.48 |
|  |  | **4** | 7.69 | 6.72 | 8.23 | 8.19 | 95 | 37.0 | 3.141 | -2.42 |
|  |  | **5** | 7.37 | 6.25 | 8.23 | 8.18 | 95 | 54.5 | - | -2.81 |
|  |  | **6** | 7.54 | 6.23 | 8.21 | 8.15 | 95 | 53.3 | 1.933 | -3.26 |
| **forest** | **light** | **1** | - | - | 8.2 | 8.36 | 99 | - | - | - |
|  |  | **2** | 7.04 | 9.66 | 8.23 | 8.32 | 99 | 326.8 | 8.371 | 6.25 |
|  |  | **3** | 7.19 | 10.10 | 8.22 | 8.35 | 103 | 286.3 | - | 6.69 |
|  |  | **4** | 7.17 | 9.23 | 8.21 | 8.33 | 102 | 306.5 | 11.417 | 4.79 |
|  |  | **5** | 7.68 | 9.73 | 8.21 | 8.38 | 104 | 565.0 | 3.358 | 4.65 |
|  |  | **6** | 7.24 | 9.21 | 8.2 | 8.27 | 103 | 310.5 | - | 4.53 |
|  | **dark** | **1** | - | - | 8.24 | 8.18 | 87 | - | - | - |
|  |  | **2** | 7.51 | 6.47 | 8.24 | 8.18 | 88 | 326.8 | 8.371 | -2.81 |
|  |  | **3** | 7.47 | 6.60 | 8.23 | 8.2 | 90 | 286.3 | - | -2.28 |
|  |  | **4** | 8.37 | 7.10 | - | - | 90 | 306.5 | 11.417 | -3.35 |
|  |  | **5** | 8.29 | 6.11 | - | - | 90 | 565.0 | 3.358 | -5.74 |
|  |  | **6** | 8.47 | 6.46 | 8.23 | 8.18 | 90 | 310.5 | - | -5.30 |

**Table S7** (continuation)

|  |  |  | **DO initial** (mg O_2_ L^-1^) | **DO final** (mg O_2_ L^-1^**)** | **pH initial** | **pH final** | **Incubation time** (min) | **Dry biomass macroalgae** (g m^-2^) | **Dry biomass macroinvertebrates** (g m^-2^) | **DO flux**  (mmol O_2_ m^-2^ h^-1^) |
| --- | --- | --- | --- | --- | --- | --- | --- | --- | --- | --- |
| **restored forest** | **light** | **1** | 7.12 | 8.75 | 8.22 | 8.32 | 94 | 314.5 | 1.520 | 4.12 |
|  |  | **2** | 6.70 | 8.06 | 8.22 | 8.24 | 95 | 410.8 | - | 3.37 |
|  |  | **3** | - | - | 8.22 | 8.27 | 95 | - | - | - |
|  |  | **4** | - | - | 8.22 | 8.28 | 97 | - | - | - |
|  |  | **5** | 7.26 | 8.95 | 8.23 | 8.32 | 98 | 238.8 | 6.315 | 4.08 |
|  |  | **6** | 7.80 | 10.37 | 8.23 | 8.49 | 100 | 503.3 | 3.817 | 6.08 |
|  | **dark** | **1** | 7.52 | 6.20 | 8.26 | 8.21 | 96 | 314.5 | 1.520 | -3.26 |
|  |  | **2** | 7.20 | 6.01 | 8.26 | 8.21 | 93 | 410.8 | - | -3.04 |
|  |  | **3** | - | - | 8.24 | 8.14 | 92 | - | - | - |
|  |  | **4** | - | - | 8.24 | 8.15 | 90 | - | - | - |
|  |  | **5** | 7.21 | 6.63 | 8.29 | 8.23 | 90 | 238.8 | 6.315 | -1.54 |
|  |  | **6** | 5.93 | 4.21 | 8.26 | 8.22 | 88 | 503.3 | 3.817 | -4.63 |
